# Dynamic changes in IFI44L and SAMHD1 mRNA levels in PBMCs are associated with treatment response in HBeAg-negative chronic hepatitis B during peg-IFN-α therapy

**DOI:** 10.3389/fmicb.2026.1976096

**Published:** 2026-09-25

**Authors:** Hao Pang, Xinglin Fu, Tianju Li, Bo Qin

**Affiliations:** 1 Department of Infectious Diseases, Chongqing Key Laboratory of Infectious Diseases and Parasitic Diseases, The First Affiliated Hospital of Chongqing Medical University, Chongqing, China; 2 Department of Infectious Diseases, Beibei Affiliated Hospital of Chongqing Medical University, Chongqing, China

**Keywords:** hepatitis B virus, IFI44L, peg-IFN-α, response, SAMHD1

## Abstract

**Objective:**

To determine whether IFI44L and SAMHD1 mRNA expression in peripheral blood mononuclear cells (PBMCs) is associated with treatment response and HBsAg clearance in patients with HBeAg-negative chronic hepatitis B (CHB) receiving pegylated interferon alpha (Peg-IFN-α).

**Methods:**

In this prospective cohort, HBeAg-negative patients with CHB received Peg-IFN-α for 48 weeks, with virological and serological responses assessed at week 48. Virological response (VR) or non-virological response (NVR) was defined according to serum HBV DNA, whereas serological response (SR) or non-serological response (NSR) was based on HBsAg loss. IFI44L and SAMHD1 mRNA levels in PBMCs collected at baseline and weeks 12 and 24 were quantified by quantitative real-time PCR. Correlation analyses were used to examine associations with changes in HBsAg and HBV DNA. Univariable and multivariable logistic regression analyses were conducted to identify factors independently associated with VR and SR, and receiver operating characteristic (ROC) curves were used to assess the discriminatory performance of the two genes.

**Results:**

Following 48 weeks of Peg-IFN-α treatment, 53.95% of patients achieved VR and 36.84% achieved SR. IFI44L and SAMHD1 expression showed distinct on-treatment patterns according to virological and serological outcomes. Higher expression of either gene at weeks 12 and 24 was associated with greater reductions in HBsAg and HBV DNA at the corresponding time points, and week 12 expression was also positively associated with the subsequent declines in both markers by week 24. In multivariable analyses, IFI44L and SAMHD1 expression at weeks 12 and 24 remained independently associated with VR and SR at week 48. ROC analysis showed greater discriminatory performance at the on-treatment assessment points, with the highest AUCs observed at week 24. At this time point, IFI44L yielded AUCs of 0.8822 for VR and 0.8445 for SR, while SAMHD1 yielded AUCs of 0.8627 and 0.8400, respectively.

**Conclusion:**

IFI44L and SAMHD1 may represent candidate dynamic on-treatment biomarkers of host interferon responsiveness during Peg-IFN-α therapy.

## Introduction

Chronic hepatitis B (CHB) remains a major worldwide health challenge and continues to contribute substantially to the development of progressive hepatic fibrosis, cirrhosis, and hepatocellular carcinoma (HCC) (Yuen et al., 2018; Danpanichkul et al., 2025). Although current antiviral therapies can effectively suppress HBV replication, they rarely eradicate the infection, and long-term consequences of persistent HBV carriage therefore remain substantial at both the clinical and socioeconomic levels (Danpanichkul et al., 2025). Current antiviral therapy for CHB is dominated by nucleos(t)ide analogs (NAs), whereas pegylated interferon alpha (Peg-IFN-α) remains an important finite-duration option for appropriately selected patients (Jeng et al., 2023). Unlike NAs, which generally require prolonged treatment and may need to be continued indefinitely in some patients, Peg-IFN-α is administered for a finite treatment course and can induce functional cure in a subset of appropriately selected patients (You et al., 2023; European Association for the Study of the Liver, 2025). According to the 2025 EASL Clinical Practice Guidelines, functional cure is defined as sustained hepatitis B surface antigen (HBsAg) loss with HBV DNA below the limit of quantification (LOQ) for at least 24 weeks after treatment cessation, with or without the development of anti-HBs (2025). Durable HBsAg clearance is clinically important because it has been associated with a reduced risk of HCC and more favorable long-term outcomes (Liang et al., 2016; Wu et al., 2025; Vittal et al., 2022). HBeAg-negative CHB is a clinically heterogeneous phase of chronic HBV infection rather than a state that uniformly reflects effective viral or immune control. Although some patients with HBeAg-negative CHB have relatively low HBsAg levels and a higher likelihood of HBsAg loss, treatment response remains highly variable across individuals (Brunetto et al., 2009; Zeng et al., 2020). HBeAg negativity itself does not necessarily indicate effective suppression of HBV replication. In many cases, alterations in the precore or basal core promoter regions prevent or markedly reduce HBeAg production without eliminating the capacity of HBV to replicate (Alexopoulou and Karayiannis, 2014; Bonino and Brunetto, 2003). Consequently, measurable or fluctuating HBV DNA may persist even in the continued absence of circulating HBeAg. Ongoing viral activity may in turn be accompanied by hepatic inflammation and progressive liver injury, with continued risk of cirrhosis and HCC development (Alexopoulou and Karayiannis, 2014; Li et al., 2024a). Long-term virological monitoring and timely initiation of antiviral treatment are therefore essential in HBeAg-negative disease (Bonino et al., 2022; Li et al., 2024a). Despite the availability of effective antiviral therapies, outcomes with Peg-IFN-α remain heterogeneous, and differences in the magnitude and kinetics of the host interferon response may contribute to this variability. Identifying early biomarkers that capture treatment-induced host responses could therefore complement conventional virological markers and improve patient stratification.

The antiviral activity of Peg-IFN-α is initiated through binding of the cytokine to the type I interferon receptor complex consisting of IFNAR1 and IFNAR2. Engagement of this receptor activates the Janus kinase (JAK)/signal transducer and activator of transcription (STAT) signaling cascade, which in turn induces a broad transcriptional program involving numerous interferon-stimulated genes (ISGs) (Sadler and Williams, 2008; Ivashkiv and Donlin, 2014). These ISGs collectively establish an antiviral state through complementary mechanisms acting at different stages of the HBV life cycle, including modification or degradation of viral nucleic acids, repression of cccDNA transcription, and interference with viral regulatory proteins (Uprichard et al., 2003; Zhao et al., 2024). For instance, APOBEC3A has been shown to facilitate cytidine deamination of HBV cccDNA, thereby promoting its degradation (Lucifora et al., 2014). Other interferon-responsive factors, including STAT1, PML, and TRIM5γ, have also been implicated in restricting HBV transcription or replication through effects on cccDNA-associated chromatin or HBx stability (Cheng et al., 2020; Tan et al., 2019). Beyond these direct effects on viral replication, differences in ISG expression in circulating immune cells and liver tissue have also been associated with the clinical response to Peg-IFN-α treatment (Han et al., 2017). These findings suggest that the magnitude and kinetics of the treatment-induced ISG response may contain clinically relevant information beyond baseline viral burden. However, biomarkers that reliably capture early host interferon responsiveness and distinguish patients with different probabilities of treatment success remain limited.

Among the large number of interferon-responsive genes implicated in HBV infection, interferon-induced protein 44-like (IFI44L) and sterile alpha motif and histidine-aspartic acid domain containing protein 1 (SAMHD1) are of particular interest because both have been linked to interferon signaling and host restriction of HBV. IFI44L has been implicated in the regulation of HBV cccDNA, with experimental evidence indicating that its antiviral activity involves modulation of NF-κB and STAT1 signaling (Nosaka et al., 2022). In HCC, reduced IFI44L expression has been associated with tumor recurrence, more advanced disease, and poorer clinical outcomes (Huang et al., 2018). IFI44L expression has also been shown to change following successful antiviral treatment in other chronic viral liver diseases, including HCV infection, supporting its potential sensitivity to treatment-related changes in host interferon activity (Brochado-Kith et al., 2021). SAMHD1 is an interferon-inducible host restriction factor that can inhibit HBV replication in hepatic cells by limiting viral DNA synthesis at the reverse-transcription step (Chen et al., 2014; Sommer et al., 2016). Its antiviral activity against HBV has been linked to its dNTPase function, which restricts the intracellular nucleotide pool required for efficient viral DNA synthesis (Sommer et al., 2016). SAMHD1 expression can also be induced by IFN-α in hepatic cells, further supporting its role as a component of the interferon-mediated antiviral response to HBV (Chen et al., 2014). Beyond chronic HBV infection, altered SAMHD1 expression has been described in other liver disorders, including HCC and metabolic dysfunction-associated steatotic liver disease (Shao et al., 2025; Yin et al., 2026). Thus, existing evidence provides a biological rationale for investigating IFI44L and SAMHD1 as interferon-responsive host factors during Peg-IFN-α treatment. Importantly, however, previous studies have focused primarily on their antiviral functions or disease-associated expression patterns, and it remains unclear whether their longitudinal expression during Peg-IFN-α therapy reflects treatment outcomes.

An important unresolved question is whether treatment-related changes in IFI44L and SAMHD1 are associated with later virological and serological outcomes in patients with HBeAg-negative CHB. Although both genes are interferon responsive and have been implicated in antiviral defense, their longitudinal mRNA expression in peripheral blood mononuclear cells (PBMCs) during Peg-IFN-α treatment has not been systematically characterized in this population. PBMC-based measurements are particularly attractive from a clinical monitoring perspective because peripheral blood can be obtained repeatedly with minimal invasiveness, making serial assessment feasible at predefined stages of treatment. This is in clear contrast to liver biopsy, which is invasive and consequently less practical for repeated longitudinal sampling. Serial measurement of ISG expression in PBMCs may therefore provide an accessible readout of systemic interferon responsiveness and, importantly, may capture treatment-induced changes that are not apparent from a single pretreatment measurement. Such dynamic information could potentially provide complementary evidence for early identification of patients more likely to benefit from Peg-IFN-α before the final treatment outcome becomes evident.

In this study, we prospectively evaluated the longitudinal expression of IFI44L and SAMHD1 mRNA in PBMCs from patients with HBeAg-negative CHB receiving Peg-IFN-α therapy. We examined whether early on-treatment changes in the expression of these two interferon-responsive genes were associated with virological response, serological response, and HBsAg clearance. We further assessed whether their longitudinal expression patterns could provide an early molecular indication of differential treatment responsiveness. By relating serial PBMC gene-expression measurements to clinical and virological outcomes, we sought to determine whether IFI44L and SAMHD1 could serve as accessible dynamic biomarkers that complement conventional markers for response stratification during Peg-IFN-α therapy. Such information may ultimately contribute to more informed patient selection and support a more individualized approach to the management of HBeAg-negative CHB.

## Methods

### Patient recruitment

We conducted a prospective observational cohort study at the Department of Infectious Diseases, the First Affiliated Hospital of Chongqing Medical University, to characterize longitudinal changes in host interferon responsiveness during Peg-IFN-α treatment in adults with HBeAg-negative chronic hepatitis B (CHB). Patients were recruited from October 2023 to October 2024. Follow-up began at enrollment and continued for 48 weeks after treatment initiation; follow-up extended beyond the recruitment period for participants enrolled later in the study. A total of 76 consecutive patients were screened and enrolled after their clinical characteristics had been assessed against the predefined eligibility criteria. All 76 enrolled patients completed the planned 48-week treatment and follow-up schedule. All patients in the prospective treatment cohort were 18–65 years of age and had not received antiviral therapy before enrollment. Clinical information and biological specimens were collected prospectively at prespecified study visits throughout follow-up. The complete study workflow, from patient screening and enrollment through initiation and continuation of Peg-IFN-α treatment, scheduled clinical and virological assessments, serial peripheral blood collection, molecular analysis of PBMC-derived samples, and determination of treatment response, is presented schematically in Supplementary Figure S1. The diagnosis of HBeAg-negative CHB and the clinical indication for antiviral therapy were determined in accordance with the 2022 recommendations jointly issued by the Chinese Society of Hepatology and the Chinese Society of Infectious Diseases (You et al., 2023). Eligibility was assessed before enrollment, and patients were included only when all prespecified inclusion criteria were fulfilled. Specifically, eligible participants were required to meet the following criteria: (1) negative serum HBeAg together with detectable anti-HBe and anti-HBc; (2) persistent serum HBsAg positivity documented for a minimum of 6 months; (3) a pretreatment serum HBV DNA level >10^4 IU/mL; (4) an ALT concentration ≥2-fold but <10-fold the upper limit of normal (ULN); and (5) no history of previous antiviral treatment. The screening process was completed before formal enrollment, and patients were excluded when any prespecified exclusion criterion was identified. Exclusion criteria comprised: (1) infection with other clinically relevant viruses, including human immunodeficiency virus (HIV) or Epstein–Barr virus (EBV); (2) concurrent inflammatory or autoimmune disorders, such as rheumatoid arthritis or autoimmune hepatitis; (3) pregnancy or lactation; (4) exposure to immunosuppressive medications during the 6 months preceding enrollment; (5) concomitant infection with hepatotropic viruses other than HBV, including hepatitis C virus (HCV) or hepatitis D virus (HDV); (6) decompensated cirrhosis or a history of malignancy; (7) alcohol intake >20 g/day in women or >30 g/day in men; (8) advanced hepatic injury or another underlying liver disease, including alcoholic liver disease or metabolic dysfunction-associated steatotic liver disease (MASLD); (9) severe psychiatric or neurological disorders, including major depressive disorder or uncontrolled epilepsy; and (10) any recognized contraindication to interferon-based treatment.

Ethical approval was obtained before commencement of the study. The study protocol was reviewed and approved by the Ethics Committee of the First Affiliated Hospital of Chongqing Medical University (Reference number: 2023–311). All participants received information regarding the study before enrollment, and written informed consent was obtained before any study-related procedure was performed. In addition to the 76 patients who received Peg-IFN-α and constituted the prospective treatment cohort, two separate reference groups were included for the baseline molecular analyses. One reference group consisted of 43 patients with untreated HBeAg-negative CHB, and the other comprised 35 healthy controls. These two reference populations served distinct purposes in the baseline molecular assessment of IFI44L and SAMHD1 expression. The untreated CHB group provided an infection-related reference representing the molecular state associated with chronic HBV infection in the absence of antiviral exposure, whereas the healthy control group provided a non-infected reference state. Considered together, these two groups allowed baseline expression of the candidate interferon-stimulated genes to be interpreted relative to both untreated chronic HBV infection and the healthy state before longitudinal changes in the Peg-IFN-α-treated cohort were assessed.

The timing of clinical, virological, and molecular measurements was prespecified before initiation of the study and remained unchanged throughout the treatment period. Serum HBsAg and HBV DNA were measured immediately before initiation of Peg-IFN-α (baseline) and every 12 weeks thereafter during the 48-week treatment course. In parallel, peripheral blood samples were collected at treatment initiation and at weeks 12 and 24. These sampling points were selected to characterize treatment-associated changes in host interferon-responsive gene expression during the early and intermediate phases of Peg-IFN-α therapy. PBMCs were isolated from the collected blood specimens, after which IFI44L and SAMHD1 transcript levels were quantified. The resulting molecular measurements were used to characterize longitudinal patterns of ISG expression and to determine whether gene-expression profiles at the prespecified early treatment time points were associated with virological and clinical outcomes assessed later in the 48-week treatment course. Treatment response was determined at week 48 according to two prespecified outcome definitions. Virological response (VR) was defined as a serum HBV DNA concentration below the lower limit of detection of the assay, corresponding to <50 IU/mL at week 48. Patients with a serum HBV DNA concentration of ≥50 IU/mL at the end of treatment were classified as having a non-virological response (NVR). Serological response (SR) was defined as the disappearance of serum HBsAg by week 48, irrespective of whether anti-HBs antibodies were detectable. HBsAg seroconversion, defined as HBsAg loss accompanied by the appearance of anti-HBs, was considered a related but distinct serological event and was assessed separately rather than being required for SR classification. This distinction was made because anti-HBs may emerge after HBsAg loss and is not required for the current definition of functional cure in contemporary AASLD/EASL treatment-endpoint framework (European Association for the Study of the Liver, 2025; Ghany et al., 2023). Participants who remained HBsAg-positive at the end of the 48-week treatment period were classified as having a non-serological response (NSR). These outcome definitions were specified before treatment-response analyses and were applied consistently throughout the study. They were used to compare pretreatment and on-treatment clinical characteristics, characterize the longitudinal behavior of IFI44L and SAMHD1 expression, examine associations between changes in HBsAg or HBV DNA and host ISG expression, and evaluate factors associated with treatment response using logistic regression and the discriminatory performance of the two candidate host-response markers using receiver operating characteristic (ROC) analysis.

### Clinical and laboratory measurements

Clinical and laboratory assessments were performed at the prespecified study visits using established laboratory methods and standardized analytical procedures. Follow-up assessments included virological, serological, biochemical, and hematological parameters. Serum HBV DNA was quantified by fluorescent real-time PCR on a Cobas Z480 platform (Roche, Switzerland), with a lower limit of detection of 50 IU/mL. HBV DNA measurements were obtained serially during follow-up to monitor virological status and were used at week 48 to classify patients into the predefined VR and NVR groups. HBV-associated serological markers, including HBsAg, anti-HBs, HBeAg, and anti-HBe, were analyzed using the Abbott Architect i2000 immunoassay system. These measurements were used to characterize serological status and to document changes in HBsAg and anti-HBs during treatment, including HBsAg loss and anti-HBs seroconversion when present. Biochemical testing was performed using an automated clinical chemistry analyzer (Cobas, Roche, and Shanghai) to assess liver-related biochemical parameters. Routine blood counts were obtained using an automated hematology analyzer (Mindray BC-6600, Shanghai), with WBC and PLT counts included among the hematological variables evaluated. The corresponding analytical platforms and standardized laboratory procedures were maintained throughout the scheduled follow-up visits to minimize variation attributable to changes in laboratory methodology or instrumentation. Clinical laboratory data were analyzed together with the molecular measurements obtained from PBMC specimens, allowing conventional virological, serological, biochemical, and hematological parameters to be evaluated alongside longitudinal changes in host gene expression during Peg-IFN-α treatment.

### RNA extraction and quantitative real-time PCR (qRT-PCR)

PBMCs obtained at the predefined treatment visits were used for analysis of IFI44L and SAMHD1 transcript expression. Total RNA was isolated from PBMC samples using TRIzol reagent according to the manufacturer’s instructions. Extracted RNA was reverse-transcribed into complementary DNA (cDNA) using a commercially available reverse-transcription kit. The resulting cDNA was used as the template for quantitative assessment of the target transcripts by a two-step real-time PCR procedure. Amplification and fluorescence acquisition were performed on a Bio-Rad CFX96 real-time PCR system (Bio-Rad, United States). Samples obtained at the pre-specified treatment time points were processed using the same analytical workflow. Each sample was analyzed in technical triplicate to improve measurement precision and reduce the influence of technical variation on the estimated expression levels. The resulting data were used to compare IFI44L and SAMHD1 expression across the study populations and across the predefined stages of Peg-IFN-α treatment. For each reaction, amplification was initiated by denaturation at 95 °C for 30 s, followed by 40 cycles of denaturation at 95 °C for 5 s and annealing/extension at 60 °C for 30 s. A melt-curve analysis was performed after amplification to assess product specificity. Further details of the experimental conditions are provided in the Supplementary methods. The primer sequences used for IFI44L and SAMHD1 amplification are listed in Supplementary Table S1. Relative mRNA abundance was calculated using the comparative cycle threshold method (2^-ΔΔCt), with GAPDH as the endogenous reference gene for normalization (Livak and Schmittgen, 2001). The normalized expression values were used in the prespecified analyses, including comparison of IFI44L and SAMHD1 expression between healthy controls and untreated patients with HBeAg-negative CHB at baseline, assessment of longitudinal changes in transcript expression during Peg-IFN-α therapy, examination of associations between gene expression and changes in serum HBsAg and HBV DNA, and evaluation of the relationships between IFI44L and SAMHD1 expression and the predefined VR and SR outcomes.

### Statistical analysis

Statistical analyses were performed using SPSS version 27.0 (IBM Corp.) and GraphPad Prism version 10.1.2 (GraphPad Software). GraphPad Prism was also used for graphical presentation of the clinical, laboratory, and molecular data. Serum HBsAg and HBV DNA concentrations were logarithmically transformed before inferential analyses because their distributions were markedly skewed. The distribution of continuous variables was assessed using the Shapiro–Wilk test. Variables with an approximately normal distribution are presented as mean ± standard deviation (SD), whereas skewed variables are summarized as median values with interquartile ranges (IQRs). The distributional characteristics of each variable were considered when selecting statistical procedures for between-group comparisons, longitudinal analyses, and correlation testing. For repeated measurements obtained from the same participants, repeated-measures analysis of variance (ANOVA) was used when the assumptions for parametric repeated-measures analysis were satisfied. When these assumptions were not met, the Friedman test was used as the corresponding nonparametric approach. These methods were applied to evaluate changes in measurements obtained at multiple pre-specified treatment time points within the same study population. For comparisons involving all three study groups, normally distributed continuous variables were analyzed using one-way ANOVA, whereas non-normally distributed continuous variables were analyzed using the Kruskal-Wallis test. Categorical variables were compared using the chi-square test or Fisher’s exact test, as appropriate. For comparisons between two independent groups, normally distributed continuous variables were analyzed using Student’s *t*-test, whereas the Mann–Whitney U test was used for variables that did not satisfy the assumption of approximate normality. Pearson correlation analysis was used to evaluate correlations between continuous variables when the assumptions for parametric correlation were satisfied, whereas Spearman rank correlation analysis was used when those assumptions were not met. Logistic regression analyses were performed separately for the two pre-specified treatment outcomes, VR and SR, to evaluate associations between candidate clinical or molecular variables and treatment response. Each candidate variable was first examined in a univariate logistic regression model. Variables associated with the outcome at *p* < 0.10 in univariate analyses were considered for multivariable modeling. Variables with established clinical relevance were also considered for inclusion irrespective of their univariate statistical significance. The final multivariable models were constructed with attention to the limited sample size and the number of observed outcome events to reduce unnecessary model complexity and the risk of overfitting. The number of covariates entered into each model was restricted with reference to the events-per-variable (EPV) principle and the number of observed events. When further reduction of candidate variables was required, priority was given to factors with greater clinical relevance. Potential collinearity between explanatory variables was also considered during model construction to avoid excessive redundancy among covariates and to preserve clinical interpretability. Formal collinearity diagnostics were additionally performed for all covariates included in the final multivariable models by calculating variance inflation factors (VIFs) and tolerance values using the same predictor sets as those used in the corresponding logistic regression analyses. HBsAg and HBV DNA were evaluated on the logarithmically transformed scale used for the regression analyses. A VIF < 5 was considered indicative of no substantial multicollinearity. The discriminatory ability of IFI44L and SAMHD1 expression for achievement of the predefined treatment outcomes was evaluated using receiver operating characteristic (ROC) curve analysis. ROC analyses were performed at the pre-specified treatment time points to assess whether gene-expression measurements obtained during treatment could discriminate between patients who achieved or failed to achieve VR or SR at week 48. The area under the ROC curve (AUC) was used as the principal measure of discrimination for each marker at each assessment point. For comparisons between paired ROC curves derived from the same participants, differences in AUCs were assessed using the DeLong test. All hypothesis tests were two-sided, and *p* < 0.05 was considered statistically significant.

## Results

### Baseline characteristics and treatment outcomes of the study population

The clinical and laboratory profiles recorded at study entry for the three study populations are presented in Table 1. The cohort included 35 healthy controls (HCs), of whom 17 were men and 18 were women; 43 untreated patients with HBeAg-negative CHB, including 19 men and 24 women; and 76 patients with HBeAg-negative CHB enrolled for Peg-IFN-α treatment, comprising 37 men and 39 women. Serum ALT and AST levels were higher in both CHB groups than in the HC group, and the difference was observed in both the untreated and treatment cohorts. No significant differences were observed among the three study groups in age, sex, WBC count, or PLT count (all overall *p* > 0.05; Table 1). In addition, direct comparison between the two CHB cohorts showed no significant differences in HBV DNA, HBsAg, ALT, AST, WBC, or PLT at baseline (all pairwise *p* > 0.05; Table 1), indicating comparable clinical and laboratory characteristics before treatment. The treatment outcomes observed over the 48-week Peg-IFN-α course are summarized in Supplementary Table S2. By the end of treatment, 41 of the 76 treated patients had achieved VR, corresponding to a response rate of 53.95%, while 28 patients met the criteria for SR, giving an overall SR rate of 36.84%. HBsAg loss became increasingly frequent as treatment progressed. At week 12, HBsAg had been cleared in 5 patients (6.58%); the corresponding numbers increased to 12 patients (15.79%) at week 24 and 18 patients (23.68%) at week 36. By week 48, a total of 28 patients (36.84%) had achieved HBsAg loss. Anti-HBs seroconversion followed a later trajectory. No patient showed seroconversion during the first 12 weeks, whereas 4 patients (5.26%) had developed anti-HBs by week 24. This number rose to 8 patients (10.53%) at week 36 and reached 20 patients (26.32%) at week 48. Thus, HBsAg loss was observed earlier than anti-HBs seroconversion during the treatment period, supporting the prespecified distinction between HBsAg loss as the operational SR endpoint and anti-HBs seroconversion as a separate serological outcome, whereas VR and SR were determined at the prespecified week 48 endpoint.

**Table 1:** Baseline characteristics in the groups.

Characteristics	HC	Untreated CHB	CHB treated with Peg-IFN-α	*p* value (Overall)	*P* value (Untreated vs. Treated CHB)
Number (n)	35	43	76		
Age (year)	41.20 ± 10.88	38.33 ± 10.42	43.50 (36.25,54.00)	0.0584	0.2475
Gender (male/female)	17/18	19/24	37/39	0.8835	0.6187
HBsAg (log10 IU/mL)	UD	3.278 (2.970,3.852)	3.037 (2.190,3.439)		0.0765
HBV DNA (log10 IU/mL)	UD	4.920 (4.551,5.238)	5.028 (4.445,5.302)		0.8310
ALT (U/L)	29.37 ± 8.318	156.1 ± 36.50^a^	148.5 (129.3,188.0)^a^	0.0043	0.7904
AST (U/L)	22.14 ± 6.656	99.00 (43.00,159.0)^a^	109.0 (45.00,141.8)^a^	0.0090	0.5996
WBC (×10^9/L)	5.669 ± 1.881	4.300 (3.900,7.530)	4.160 (3.158,5.978)	0.2736	0.6022
PLT (×10^9/L)	164.6 ± 46.59	169.0 (136.0,202.0)	155.1 ± 62.08	0.1675	0.0846

HBsAg: hepatitis B surface antigen; AST: aspartate aminotransferase; ALT: alanine aminotransferase; WBC: white blood cells; PLT: platelet; HC: Healthy control; Data are expressed as mean ± standard deviation (SD) for normally distributed variables or median (interquartile range) for non-normally distributed variables. Overall p values represent comparisons among the three study groups. Pairwise *p* values represent direct comparisons between untreated CHB patients and CHB patients treated with Peg-IFN-α. a Indicates a statistically significant difference compared with the HC group (*p* < 0.05). UD, undetected.

### Reduced baseline expression of IFI44L and SAMHD1 in PBMCs from untreated CHB patients

Baseline PBMC expression of IFI44L and SAMHD1 was compared between untreated patients with HBeAg-negative CHB and healthy controls to determine whether the two transcripts differed before antiviral treatment. Both genes were quantified by qRT-PCR using RNA extracted from PBMCs. IFI44L expression was significantly lower in untreated CHB patients than in HCs (*p* = 0.0030; Figure 1A). SAMHD1 showed the same direction of difference, with a more marked reduction in the untreated CHB group (*p* = 0.0001; Figure 1B). Overall, both IFI44L and SAMHD1 showed significantly lower baseline expression in PBMCs from untreated patients with HBeAg-negative CHB than in HCs.

**Figure 1:**
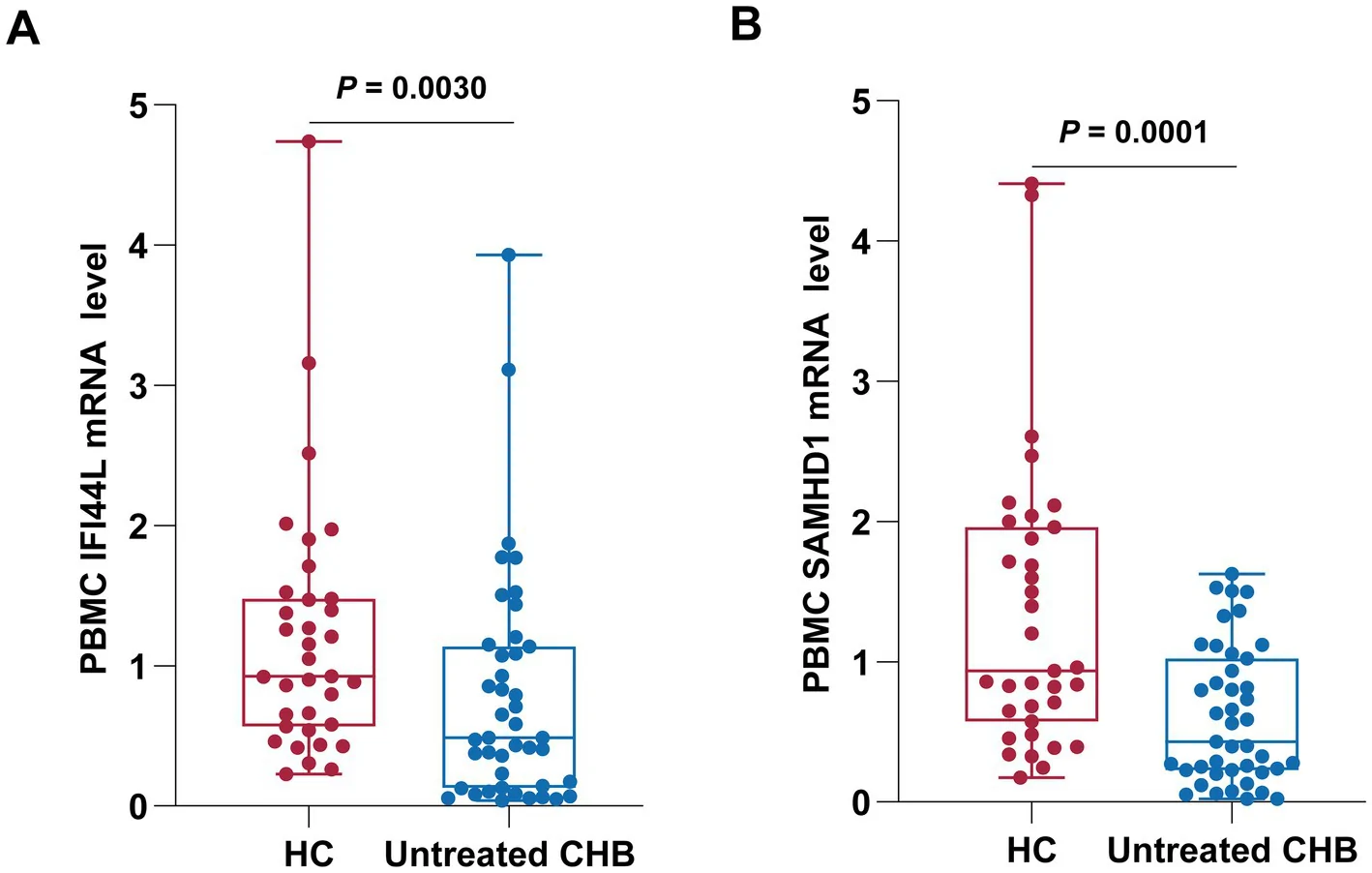
IFI44L and SAMHD1 mRNA expression in PBMCs of untreated CHB patients and healthy controls. IFI44L **(A)** and SAMHD1 **(B)** mRNA levels in PBMCs were analyzed by qRT-PCR. Expression levels were calculated using the comparative cycle threshold method (2^-ΔΔCt), with GAPDH as the reference gene. *p* < 0.05 was considered statistically significant.

### Clinical and laboratory characteristics according to treatment response

Baseline and early on-treatment clinical and laboratory variables were then compared according to the treatment outcomes observed at week 48. For the analysis based on VR, HBsAg levels were consistently lower in patients who ultimately achieved VR than in those classified as NVR. This difference was present before Peg-IFN-α treatment and remained evident at both week 12 and week 24, with *p* < 0.0001 at all three time points (Supplementary Figure S2 and Table 2). A similar distribution was observed for serum HBV DNA. Patients who achieved VR had lower HBV DNA concentrations at baseline (*p* = 0.0001), week 12 (*p* < 0.0001), and week 24 (*p* < 0.0001; Supplementary Figure S2 and Table 2). ALT and AST showed a different temporal pattern from that of the viral markers. ALT did not differ significantly between the VR and NVR groups at baseline or week 12, with *p* values >0.05 at both time points (Supplementary Figure S2 and Table 2). At week 24, however, ALT levels were significantly higher in the VR group (*p* = 0.0165), and AST showed a similar difference (*p* = 0.0174). In contrast, WBC and PLT counts remained comparable between the two groups at baseline, week 12, and week 24, and none of these comparisons reached statistical significance (all *p* > 0.05; Supplementary Figure S2 and Table 2).

**Table 2:** Comparison of clinical characteristics between virological response (VR) group and non-virological response (NVR) group.

characteristics	All	0w	NVR group (*n* = 35)	*p* value	12w	NVR group (*n* = 35)	*P* value	24w	NVR group (*n* = 35)	*P* value
	(*n* = 76)	VR group (*n* = 41)			VR group (*n* = 41)			VR group (*n* = 41)		
HBsAg (log10 IU/mL)	3.037 (2.190,3.439)	2.212 (1.817,3.076)	3.305 (3.058,3.547)	**<0.0001**	1.734 (0.828,2.882)	3.186 (2.889,3.369)	**<0.0001**	1.104 (−1.398 ,2.040)	2.967 (2.326,3.292)	**<0.0001**
HBV DNA (log10 IU/mL)	5.028 (4.445,5.302)	4.504 (4.374,5.157)	5.214 (4.937,5.417)	**0.0001**	3.714 (1.699,4.180)	4.723 (4.455,4.997)	**<0.0001**	1.734 (1.699 ,3.482)	4.479 (4.013,4.724)	**<0.0001**
ALT (U/L)	148.5 (129.3,188.0)	154.0 (129.5,195.0)	139.0 (127.0,185.0)	0.4443	70.00 (34.00,129.0)	57.00 (40.00,102.0)	0.5703	36.00 (23.00 ,57.50)	25.00 (20.00,32.00)	**0.0165**
AST (U/L)	109.(45.00 ,141.8)	112.0(44.50 ,149.0)	108.0 (45.00,141.0)	0.8826	42.00 (26.50,103.5)	35.00 (25.00,56.00)	0.0548	36.00 (22.50 ,58.50)	24.00 (18.00,42.00)	**0.0174**
PLT (×10^9/L)	155.1± 62.08	168.0± 70.19	139.8± 47.56	0.0850	167.2± 70.54	162.6± 59.89	0.7566	154.6± 72.51	171.7± 66.71	0.4260
WBC (×10^9/L)	4.160 (3.158,5.978)	3.430 (3.210,6.285)	4.279 ± 1.546	0.3358	3.610 (3.145,4.935)	4.800± 1.348	0.0906	4.050 (3.315 ,6.055)	5.101± 1.877	0.2837

HBsAg, hepatitis B surface antigen; ALT, alanine aminotransferase; AST, aspartate aminotransferase; WBC, white blood cells; PLT, platelet; VR, virological response; NVR, non-virological response; Data are expressed as mean ± standard deviation (SD) for normally distributed variables or median (interquartile range) for non-normally distributed variables. Bold values are statistically significant *p* < 0.05.

The same parameters were assessed using SR as the predefined treatment endpoint. HBsAg concentrations were lower in patients who achieved SR than in those classified as NSR at each of the three assessment points. The differences were statistically significant at baseline, week 12, and week 24, with *p* < 0.0001 at all time points (Supplementary Figure S3 and Table 3). Serum HBV DNA followed the same overall pattern, with lower levels in the SR group at baseline (*p* = 0.0013), week 12 (*p* = 0.0004), and week 24 (*p* < 0.0001; Supplementary Figure S3 and Table 3). No significant differences between the SR and NSR groups were observed for ALT, AST, WBC, or PLT at any of the examined time points, with all *p* values >0.05 (Supplementary Figure S3 and Table 3). Among the clinical and laboratory variables assessed, the most consistent between-group differences according to treatment response were observed for serum HBsAg and HBV DNA.

**Table 3:** Comparison of clinical characteristics between serological response (SR) group and non-serological response (NSR) group.

characteristics	All	0w	NSR group (*n* = 48)	*P* value	12w		*P* value	24w	NSR group (*n* = 48)	*P* value
	(*n* = 76)	SR group (*n* = 28)			SR group (*n* = 28)	NSR group (*n* = 48)		SR group (*n* = 28)		
HBsAg (log10 IU/mL)	3.037 (2.190,3.439)	1.939 (1.358,2.678)	3.300 (2.942,3.522)	**<0.0001**	1.465 (0.069,1.941)	3.085 (2.730,3.309)	**<0.0001**	0.907 (−1.492 ,1.530)	2.953 (2.211,3.265)	**<0.0001**
HBV DNA (log10 IU/mL)	5.028 (4.445,5.302)	4.465 (4.374,5.148)	5.140 (4.763,5.358)	**0.0013**	3.506 (1.699,4.201)	4.531 (3.835,4.984)	**0.0004**	1.712 (1.699, 3.216)	4.185 (3.537,4.697)	**<0.0001**
ALT (U/L)	148.5 (129.3,188.0)	163.2± 41.37	141.5 (127.8,187.3)	0.6511	68.50 (38.25,133.0)	64.50 (38.00,106.3)	0.4163	36.50 (23.50, 57.00)	26.00 (19.00,45.50)	0.2339
AST (U/L)	109 (45.00,141.8)	111.0± 57.67	108.0 (44.00,139.3)	0.4232	44.50 (26.75,106.0)	35.00 (26.00,84.50)	0.2772	40.86± 18.49	27.00 (20.25,37.00)	0.6494
PLT (10^9/L)	155.1± 62.08	154.0± 70.37	155.7± 57.48	0.7340	165.5 ±72.04	164.9± 62.10	0.9706	147.4± 71.83	171.2 ±68.07	0.1618
WBC (10^9/L)	4.160 (3.158,5.978)	3.695 (3.168,5.353)	4.629± 1.719	0.5328	3.595 (3.113,4.663)	4.733± 1.457	0.1638	3.880 (3.405 ,6.108)	4.765 (3.675,6.233)	0.2896

HBsAg, hepatitis B surface antigen; ALT, alanine aminotransferase; AST, aspartate aminotransferase; WBC, white blood cells; PLT, platelet; SR, serological response; NSR, non-serological response; Data are expressed as mean ± standard deviation (SD) for normally distributed variables or median (interquartile range) for non-normally distributed variables. Bold values are statistically significant *p* < 0.05.

### Longitudinal changes in IFI44L and SAMHD1 expression during peg-IFN-α treatment

PBMCs obtained before treatment and at weeks 12 and 24 were used to follow the expression of IFI44L and SAMHD1 throughout the early phase of Peg-IFN-α therapy. The longitudinal analyses were stratified according to the treatment response determined at week 48. For each predefined endpoint, expression at the two on-treatment time points was first compared with the corresponding baseline level within the same response group, followed by comparisons between patients who achieved the endpoint and those who did not. This design allowed the temporal behavior of the two transcripts to be evaluated in relation to both the VR/NVR classification and the SR/NSR classification.

For the patients who ultimately achieved VR, IFI44L expression was significantly higher than baseline at week 12 (*p* = 0.0151) and remained significantly elevated at week 24 (*p* < 0.0001; Figure 2A). SAMHD1 followed a comparable trajectory. Its expression was significantly elevated relative to baseline at week 12 (*p* = 0.0358) and remained significantly higher than baseline at week 24 (*p* < 0.0001; Figure 2D). Thus, both transcripts showed significant increases from baseline at weeks 12 and 24 among patients who achieved VR. No significant longitudinal change was observed in the NVR group. IFI44L levels at weeks 12 and 24 were not significantly different from baseline, and SAMHD1 likewise remained statistically unchanged over the same interval. For both genes, all within-group comparisons in NVR patient’s yielded *p* > 0.05 (Figure 2B and Figure 2E). Between-group comparisons showed that IFI44L expression was significantly higher in the VR group than in the NVR group at week 12 (*p* = 0.0145) and remained significantly higher at week 24 (*p* < 0.0001; Figure 2C). SAMHD1 showed the same directional pattern, with significantly greater expression in the VR group at week 12 and a significant between-group difference that remained evident at week 24 (*p* < 0.0001 for both comparisons; Figure 2F). Thus, significant between-group differences in both genes were detectable by week 12 and persisted at week 24.

**Figure 2:**
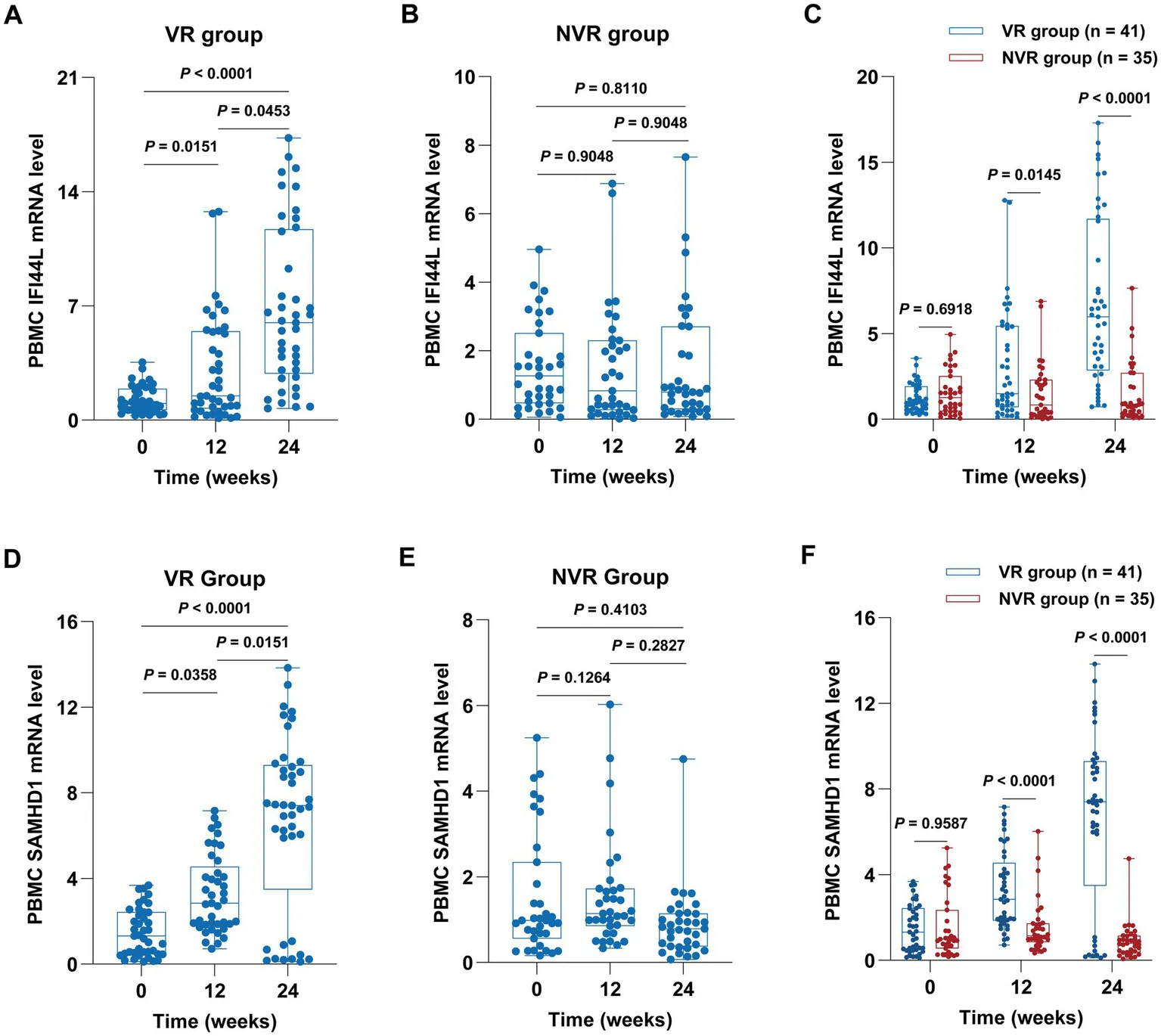
Dynamic changes in IFI44L and SAMHD1 mRNA levels in the VR and NVR groups. **(A,B)** IFI44L mRNA levels during therapy in the VR and NVR groups. **(D,E)** SAMHD1 mRNA levels during therapy in the VR and NVR groups. **(C,F)** IFI44L and SAMHD1 mRNA levels in the VR and NVR groups were compared at weeks 0, 12, and 24 of therapy. IFI44L and SAMHD1 mRNA levels in PBMCs were quantified by qRT-PCR using the 2^-ΔΔCt method, with GAPDH as the reference gene. Values are presented as median (interquartile range). *p* < 0.05 was considered statistically significant.

The longitudinal analyses were then repeated after redefining treatment outcome according to SR. Among patients who achieved SR, IFI44L expression rose significantly from baseline by week 12 (*p* = 0.0001) and remained significantly elevated at week 24 (*p* < 0.0001; Figure 3A). SAMHD1 also increased during treatment. Its expression was higher than baseline at week 12 (*p* = 0.0098) and remained significantly higher than baseline at week 24 (*p* < 0.0001; Figure 3D). Both genes therefore showed significant increases from baseline at each on-treatment sampling point in the SR group. A different pattern was observed in the NSR group, in which neither IFI44L nor SAMHD1 showed a significant longitudinal change over the assessed treatment period. For both transcripts, expression at weeks 12 and 24 remained comparable with the corresponding baseline measurements, with all *p* values >0.05 (Figure 3B and Figure 3E). Between-group comparisons further showed that IFI44L expression was significantly higher in the SR group than in the NSR group at week 12 (*p* = 0.0005) and remained significantly higher at week 24 (*p* < 0.0001; Figure 3C). SAMHD1 exhibited the same pattern, with higher expression in the SR group at both week 12 and week 24; the corresponding comparisons were significant at each time point (*p* < 0.0001 for both; Figure 3F). Thus, both genes showed significant between-group differences at weeks 12 and 24 according to the SR outcome.

**Figure 3:**
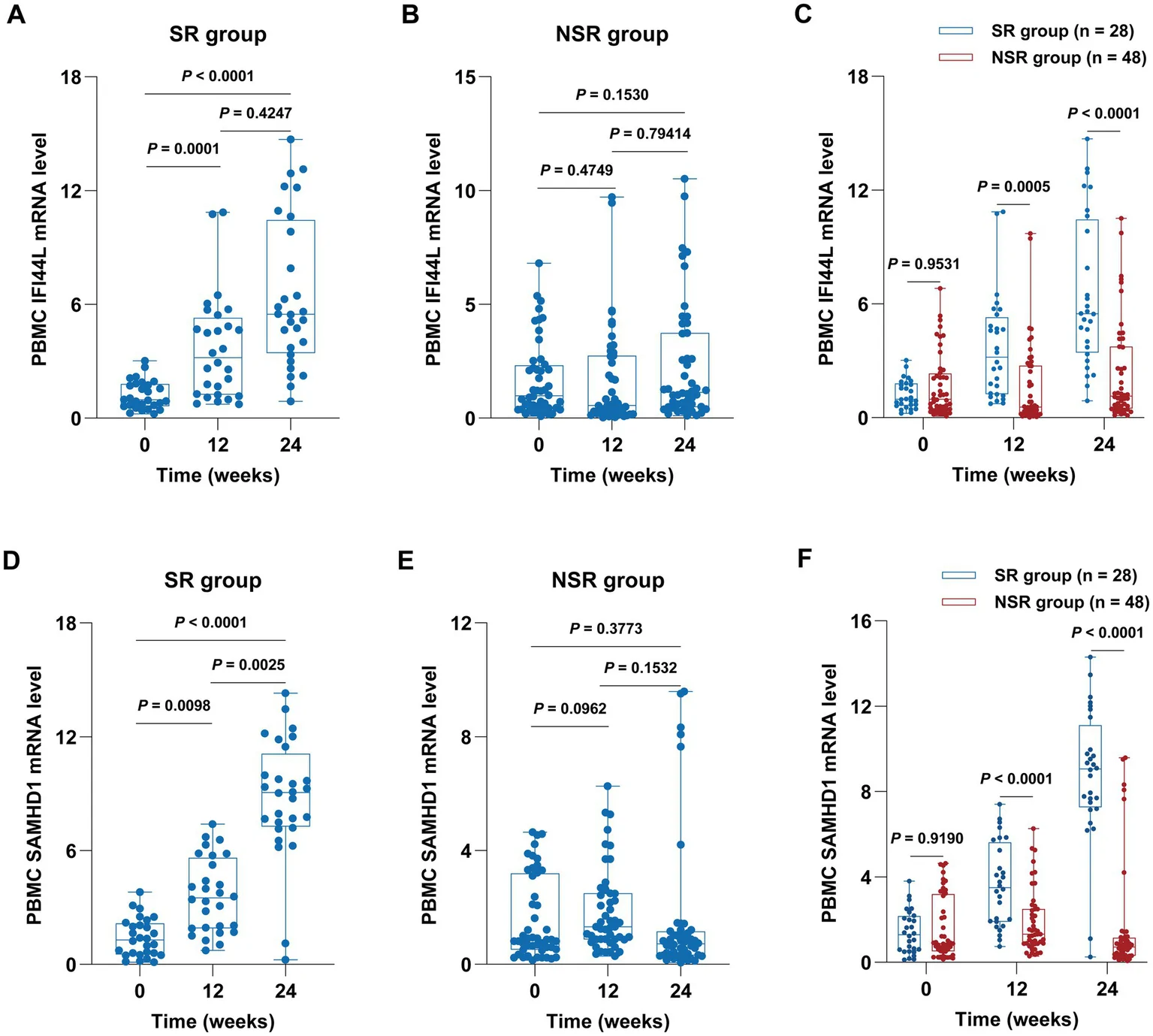
Dynamic changes in IFI44L and SAMHD1 mRNA levels in the SR and NSR groups. **(A,B)** IFI44L mRNA levels during therapy in the SR and NSR groups. **(D,E)** SAMHD1 mRNA levels during therapy in the SR and NSR groups. **(C,F)** IFI44L and SAMHD1 mRNA levels in the SR and NSR groups were compared at weeks 0, 12, and 24 of therapy. IFI44L and SAMHD1 mRNA levels in PBMCs were quantified by qRT-PCR using the 2^-ΔΔCt method, with GAPDH as the reference gene. Values are presented as median (interquartile range). *p* < 0.05 was considered statistically significant.

Overall, patients who achieved either VR or SR showed significant increases in IFI44L and SAMHD1 expression from baseline at weeks 12 and 24, whereas no significant longitudinal changes were observed in the corresponding NVR and NSR groups. At both on-treatment assessment points, IFI44L and SAMHD1 expression was also significantly higher in responder groups than in their respective non-responder groups.

### Association of IFI44L and SAMHD1 expression with declines in HBsAg and HBV DNA

The relationship between the two PBMC-derived transcripts and changes in circulating viral markers was evaluated next. IFI44L and SAMHD1 expression measured at weeks 12 and 24 was correlated with the extent of HBsAg reduction recorded at the corresponding time point. At week 12, higher IFI44L expression was associated with a greater reduction in HBsAg (*r* = 0.2560, *p* = 0.0256; Figure 4A). A significant positive relationship was also observed for SAMHD1, with its expression level correlating with the magnitude of HBsAg decline (*r* = 0.3113, *p* = 0.0062; Figure 4B). At week 24, these positive associations remained evident for both transcripts. IFI44L expression was positively correlated with the extent of HBsAg reduction (*r* = 0.3756, *p* = 0.0008; Figure 4C), while SAMHD1 showed a significant positive correlation with HBsAg decline (*r* = 0.4033, *p* = 0.0003; Figure 4D). We next examined whether gene expression at week 12 was associated with HBsAg decline at a later treatment time point. Week 12 IFI44L expression was significantly and positively associated with the reduction in HBsAg observed at week 24 (*r* = 0.4246, *p* = 0.0003; Figure 4E). The corresponding analysis for SAMHD1 produced a significant positive correlation as well (*r* = 0.3466, *p* = 0.0022; Figure 4F). Thus, expression of both transcripts was associated not only with concurrent HBsAg decline but also with subsequent HBsAg reduction over the following 12 weeks.

**Figure 4:**
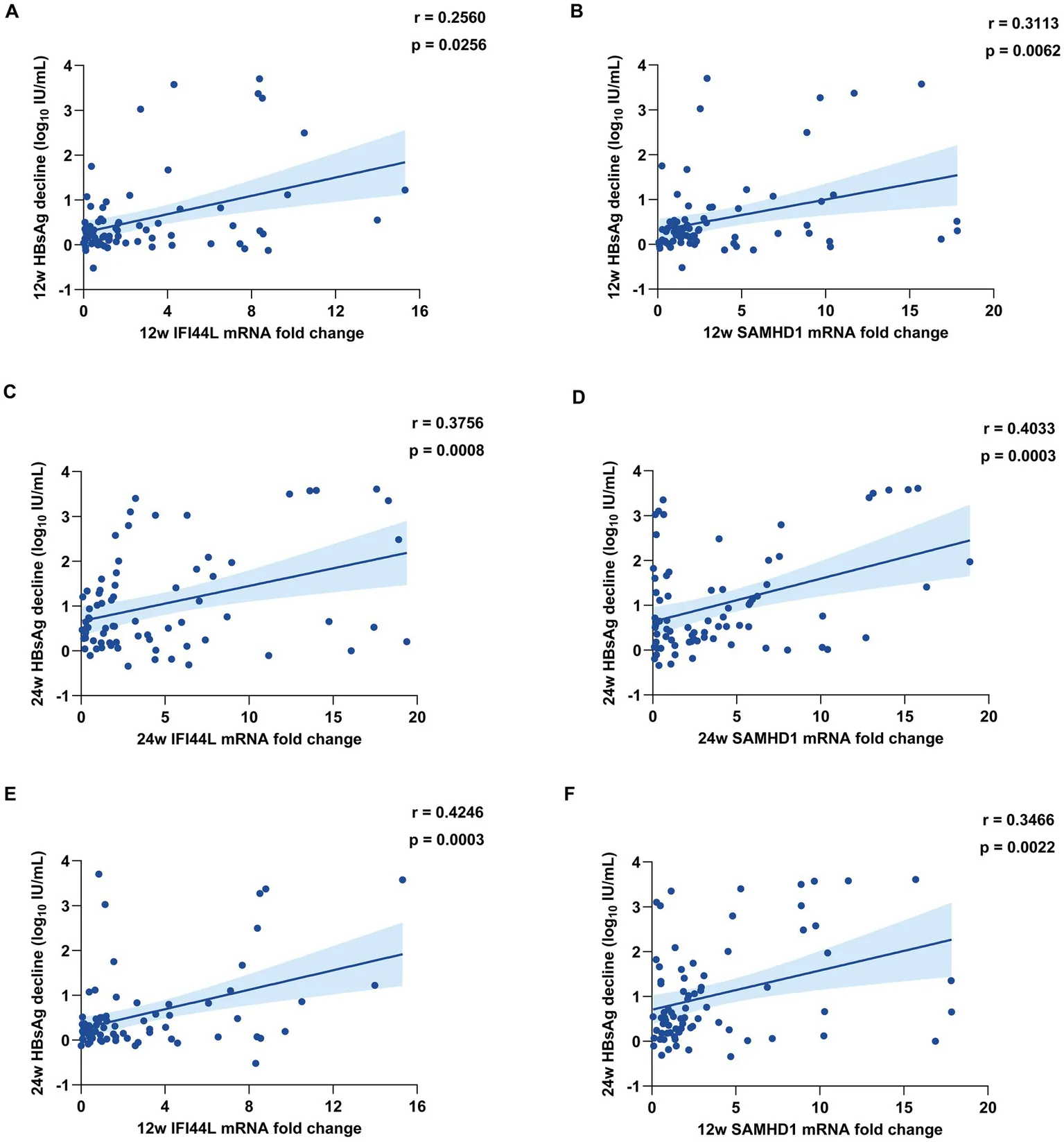
Correlations of IFI44L and SAMHD1 mRNA levels with HBsAg reduction during Peg-IFN-α therapy. **(A,B)** Correlations of IFI44L and SAMHD1 mRNA levels with HBsAg reduction at week 12. **(C,D)** Correlations of IFI44L and SAMHD1 mRNA levels with HBsAg reduction at week 24. **(E,F)** Correlations of IFI44L and SAMHD1 mRNA levels at week 12 with HBsAg reduction at week 24. IFI44L and SAMHD1 mRNA levels in PBMCs were quantified by qRT-PCR using the 2^-ΔΔCt method, with GAPDH as the reference gene. HBsAg levels were log10 transformed prior to analysis, and the magnitude of HBsAg reduction was calculated as the baseline log10-transformed level minus the corresponding on-treatment level. Correlations were assessed using Spearman’s rank correlation analysis. The correlation coefficient (r) and two-tailed *p* values were calculated. *p* < 0.05 was considered statistically significant.

A parallel analysis was carried out using HBV DNA decline as the outcome measure. At week 12, IFI44L expression was positively correlated with the reduction in HBV DNA measured at the same visit (*r* = 0.3132, *p* = 0.0059; Figure 5A). SAMHD1 showed a similar association, with higher transcript levels corresponding to a greater concurrent decrease in HBV DNA (*r* = 0.2934, *p* = 0.0101; Figure 5B). By week 24, significant positive correlations remained evident for both genes. The extent of HBV DNA reduction correlated with IFI44L expression (*r* = 0.4262, *p* = 0.0004; Figure 5C) and with SAMHD1 expression (*r* = 0.4081, *p* = 0.0003; Figure 5D). We then assessed whether week 12 expression was associated with the subsequent change in HBV DNA at week 24. IFI44L expression at week 12 was significantly associated with the subsequent reduction in HBV DNA at week 24 (*r* = 0.3674, *p* = 0.0015; Figure 5E). SAMHD1 yielded a comparable result, with week 12 expression positively correlated with the decrease in HBV DNA recorded at week 24 (*r* = 0.3932, *p* = 0.0004; Figure 5F). Taken together, these analyses showed that both transcripts were associated with concurrent HBV DNA decline and with the subsequent reduction observed at week 24 when expression was assessed at week 12.

**Figure 5:**
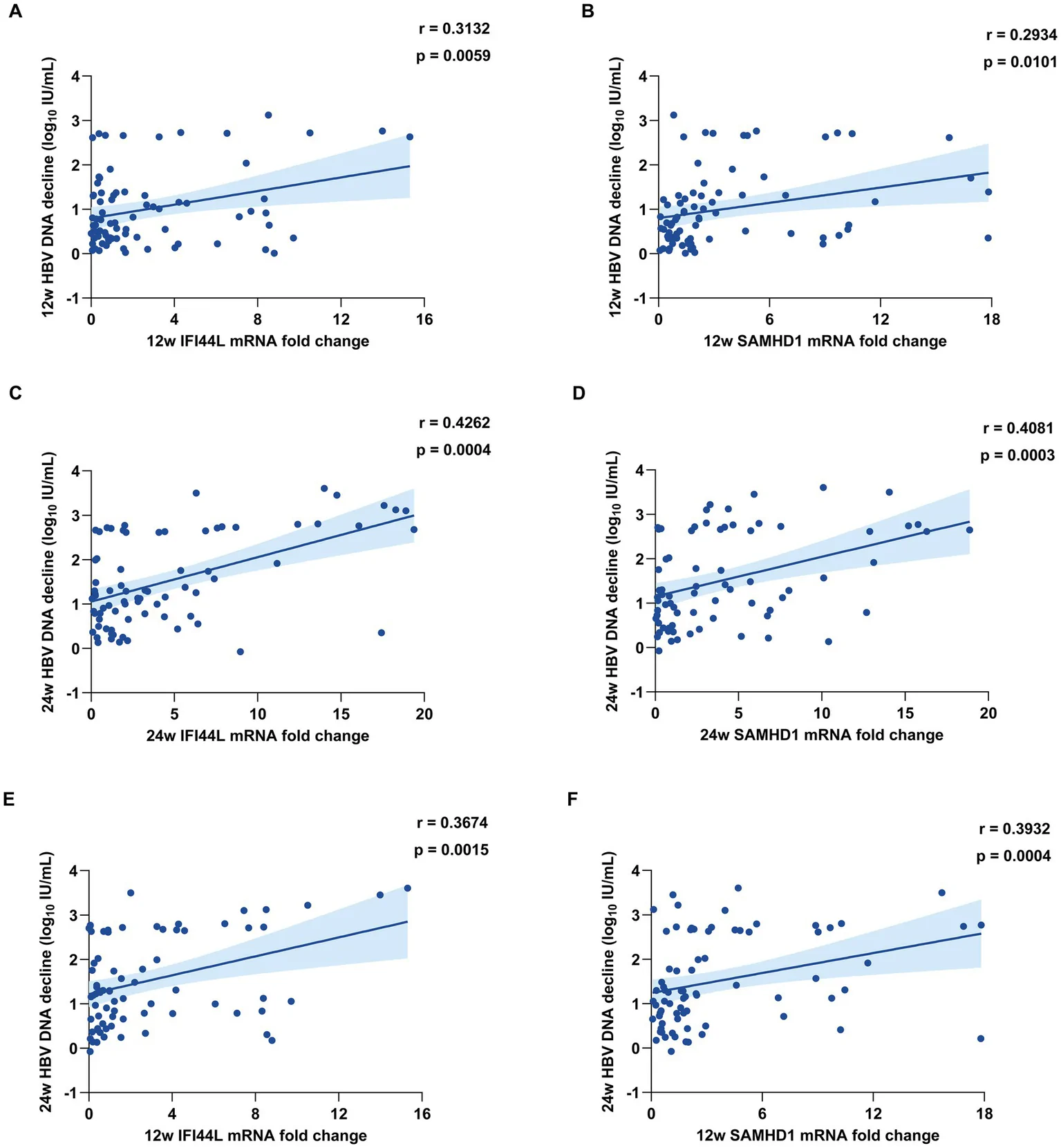
Correlations of IFI44L and SAMHD1 mRNA levels with HBV DNA reduction during Peg-IFN-α therapy. **(A,B)** Correlations of IFI44L and SAMHD1 mRNA levels with HBV DNA reduction at week 12. **(C,D)** Correlations of IFI44L and SAMHD1 mRNA levels with HBV DNA reduction at week 24. **(E,F)** Correlations of IFI44L and SAMHD1 mRNA levels at week 12 with HBV DNA reduction at week 24. IFI44L and SAMHD1 mRNA levels in PBMCs were quantified by qRT-PCR using the 2^-ΔΔCt method, with GAPDH as the reference gene. HBV DNA levels were log10 transformed prior to analysis, and the magnitude of HBV DNA reduction was calculated as the baseline log10-transformed level minus the corresponding on-treatment level. Correlations were assessed using Spearman’s rank correlation analysis. The correlation coefficient (r) and two-tailed *p* values were calculated. *p* < 0.05 was considered statistically significant.

### Factors independently associated with virological and serological response

To identify variables associated with the two prespecified treatment outcomes, separate logistic regression analyses were conducted for VR and SR. For each endpoint, variables were first examined individually in univariate models, after which multivariable models were constructed to determine whether the observed associations persisted after simultaneous adjustment for the other candidate factors included at the respective treatment time point.

For the VR endpoint, the baseline univariate analysis identified HBsAg and HBV DNA as significant factors. At week 12, HBsAg, HBV DNA, IFI44L, and SAMHD1 were significantly associated with VR at week 48. By week 24, six variables were significantly associated with VR in the univariate analysis, including HBsAg, HBV DNA, ALT, AST, IFI44L, and SAMHD1 (Supplementary Table S3). After multivariable adjustment, HBV DNA was the only variable that remained significantly associated with VR at baseline, with an adjusted odds ratio (aOR) of 0.094 (*p* = 0.0043). At week 12, lower HBV DNA remained associated with a greater likelihood of VR (aOR = 0.047, *p* = 0.0018). IFI44L expression was also independently and positively associated with VR (aOR = 1.578, *p* = 0.0289), as was SAMHD1 expression (aOR = 1.746, *p* = 0.0243). Thus, HBV DNA, IFI44L, and SAMHD1 were independently associated with VR at week 48 in the multivariable model based on week 12 measurements (Supplementary Table S3). At week 24, HBV DNA remained inversely associated with VR (aOR = 0.097, *p* = 0.0054), whereas IFI44L and SAMHD1 retained independent positive associations with the outcome, with aORs of 2.267 (*p* = 0.0019) and 2.563 (*p* = 0.0083), respectively. Accordingly, HBV DNA, IFI44L, and SAMHD1 remained independently associated with VR in the week 24 multivariable model (Supplementary Table S3).

An analogous series of regression analyses was then performed with SR as the outcome. At baseline, HBsAg and HBV DNA were significantly associated with SR in the univariate analyses. By week 12, four variables met the significance criterion, including HBsAg, HBV DNA, IFI44L, and SAMHD1. The same four variables were significantly associated with SR at week 24 (Supplementary Table S4). Multivariable analysis produced a smaller set of variables independently associated with SR. At baseline, HBsAg retained an inverse association with the serological endpoint, with an aOR of 0.064 (*p* = 0.0002). At week 12, HBsAg remained inversely associated with SR (aOR = 0.168, *p* = 0.0001), whereas IFI44L and SAMHD1 were independently and positively associated with SR, with aORs of 1.443 (*p* = 0.0053) and 1.574 (*p* = 0.0262), respectively (Supplementary Table S4). At week 24, HBsAg remained inversely associated with SR (aOR = 0.137, *p* = 0.0056), while IFI44L and SAMHD1 retained independent positive associations with the outcome, with aORs of 1.616 (*p* = 0.0055) and 2.162 (*p* = 0.0026), respectively (Supplementary Table S4). Thus, HBsAg, IFI44L, and SAMHD1 were independently associated with SR at week 48 in the multivariable model based on week 24 measurements. Collinearity diagnostics were performed for the final multivariable models at baseline, week 12, and week 24. All VIF values were ≤2.24, and the corresponding tolerance values were ≥0.4464 (Supplementary Table S5), providing no evidence of substantial multicollinearity among the covariates included in the final models.

### Discriminatory performance of IFI44L and SAMHD1 for treatment response

The discriminatory value of IFI44L and SAMHD1 expression for treatment outcomes was evaluated using receiver operating characteristic (ROC) curve analysis. Measurements obtained at baseline, week 12, and week 24 were examined separately to determine the ability of each marker to discriminate patients according to their VR or SR status at week 48.

For VR, IFI44L showed minimal discriminatory value before treatment, with a baseline AUC of 0.5268 (95% CI, 0.3914–0.6623). At week 12, the AUC increased to 0.6627 (95% CI, 0.5408–0.7846), reaching 0.8822 at week 24 (95% CI, 0.8089–0.9556). The week 24 measurement therefore provided the strongest discrimination among the three assessment points (Figure 6A and Supplementary Table S6). At the optimal cutoff of 3.7326, the sensitivity was 75.30% and the specificity was 91.40% (Supplementary Table S6). The analysis based on SR showed a similar temporal pattern for IFI44L. Its baseline AUC was 0.5045 (95% CI, 0.3759–0.6330), indicating little discrimination between patients according to their SR status at week 48. The AUC increased to 0.8021 at week 12 (95% CI, 0.7060–0.8982) and reached 0.8445 at week 24 (95% CI, 0.7583–0.9307), again with the strongest discrimination observed at week 24 (Figure 6B and Supplementary Table S6). With a cutoff of 1.6029, the sensitivity and specificity were 85.40 and 82.50%, respectively (Supplementary Table S6). SAMHD1 displayed a comparable pattern for VR. At baseline, the AUC was 0.5038 (95% CI, 0.3696–0.6381). At week 12, the AUC increased to 0.8272 (95% CI, 0.7319–0.9225) and reached 0.8627 at week 24 (95% CI, 0.7740–0.9514), with the strongest discrimination observed at week 24 (Figure 6C and Supplementary Table S6). At the cutoff value of 5.3252, SAMHD1 yielded a sensitivity of 75.60% and a specificity of 92.10% (Supplementary Table S6). For SR, the SAMHD1 AUC was 0.5074 (95% CI, 0.3727–0.6421) at baseline, increased to 0.7790 at week 12 (95% CI, 0.6740–0.8840), and reached 0.8400 at week 24 (95% CI, 0.7444–0.9357). The strongest discrimination was likewise observed at week 24 (Figure 6D and Supplementary Table S6). At a cutoff of 2.8310, the sensitivity was 82.10% and the specificity was 81.20% (Supplementary Table S6).

**Figure 6:**
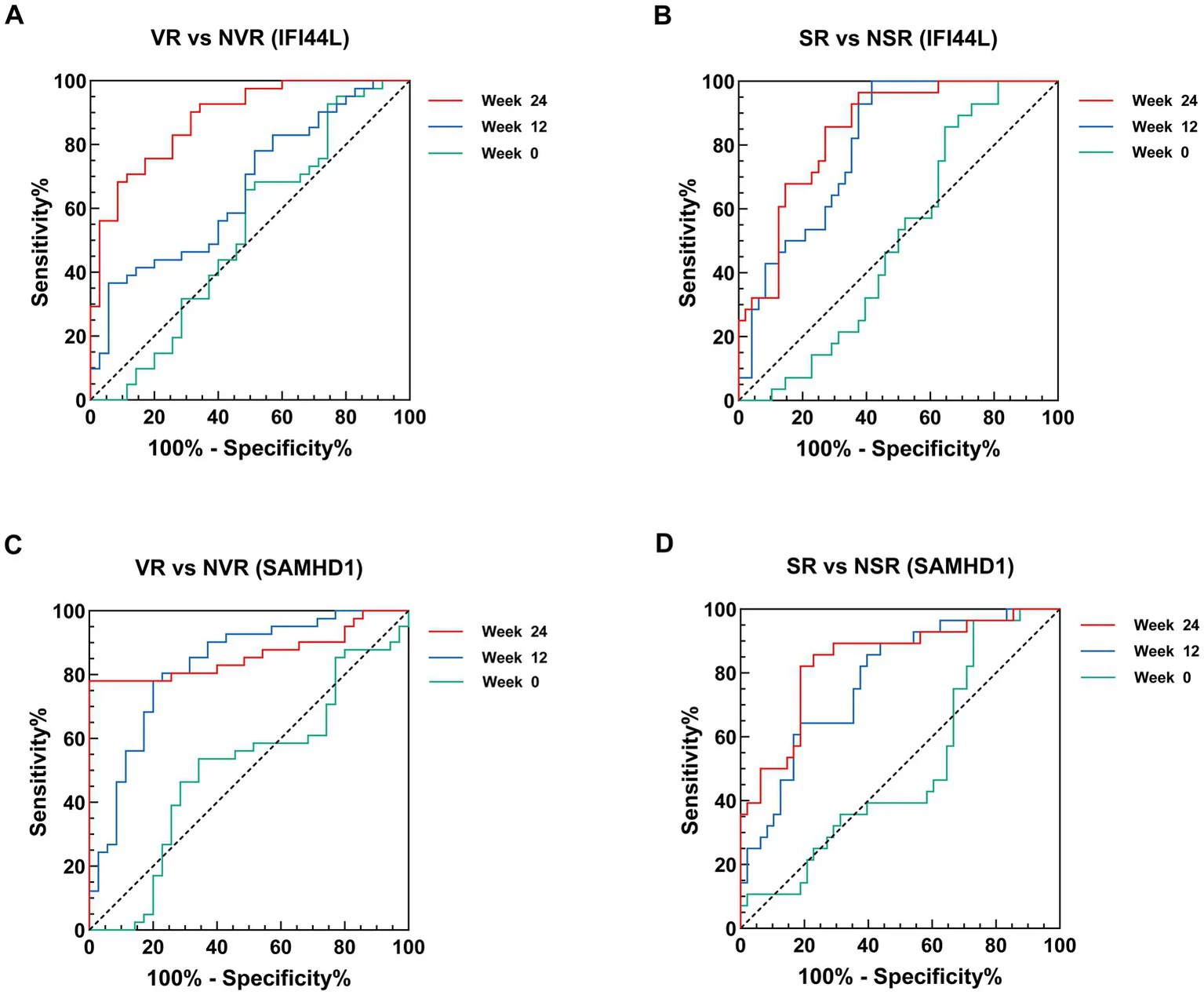
Receiver operating characteristic (ROC) curves for the discriminatory performance of IFI44L and SAMHD1 during Peg-IFN-α therapy. **(A,B)** ROC curves for IFI44L mRNA levels at weeks 0, 12, and 24 to discriminate VR and SR at week 48. **(C,D)** ROC curves for SAMHD1 mRNA levels at weeks 0, 12, and 24 to discriminate VR and SR at week 48. The Youden index was used to determine the cohort-derived cutoff values. AUC, area under the curve.

Overall, both IFI44L and SAMHD1 showed little discriminatory value at baseline, whereas their ability to discriminate patients according to VR and SR status at week 48 increased after initiation of Peg-IFN-α therapy. Meaningful discrimination was already evident at week 12, and the highest AUC values for both VR and SR were observed at week 24.

### Landmark analysis of IFI44L and SAMHD1 for treatment response

To further examine the temporal relationship between on-treatment biomarker measurements and treatment outcomes, landmark analyses were performed at weeks 12 and 24. For the serological outcome, patients who had already achieved HBsAg loss by the corresponding landmark time point were excluded. At week 12, 5 patients had already achieved HBsAg loss and were therefore excluded, leaving 71 patients for analysis. Among these patients, 23 achieved HBsAg loss by week 48, whereas 48 remained HBsAg-positive. At week 24, 12 patients had already achieved HBsAg loss and were excluded, leaving 64 patients, of whom 16 achieved HBsAg loss by week 48 and 48 remained HBsAg-positive. The landmark ROC analysis showed that the discriminatory performance of IFI44L and SAMHD1 remained evident after excluding patients who had already achieved HBsAg loss at the corresponding time point. At week 12, the AUCs for IFI44L and SAMHD1 were 0.7899 (95% CI, 0.6873–0.8924; *p* < 0.0001) and 0.7319 (95% CI, 0.6131–0.8506; *p* = 0.0017), respectively. At week 24, the corresponding AUCs were 0.8110 (95% CI, 0.7064–0.9156; *p* = 0.0004) for IFI44L (Supplementary Figure S4 and Supplementary Table S7) and 0.8065 (95% CI, 0.6765–0.9366; *p* = 0.0005) for SAMHD1 (Supplementary Figure S4 and Supplementary Table S7). Although these AUC estimates were lower than those obtained in the original ROC analyses, both transcripts continued to show significant discriminatory performance at weeks 12 and 24.

A corresponding landmark analysis was also performed for the virological outcome. Eleven patients had achieved HBV DNA < 50 IU/mL by week 12 and were excluded from the week 12 analysis, leaving 65 patients. At week 24, 26 patients had achieved HBV DNA < 50 IU/mL and were excluded, leaving 50 patients for the week 24 analysis. The AUC for IFI44L was 0.6419 (95% CI, 0.5055–0.7783; *p* = 0.0499) at week 12 and 0.7939 (95% CI, 0.6613–0.9264; *p* = 0.0014) at week 24 (Supplementary Figure S4 and Supplementary Table S7). For SAMHD1, the corresponding AUCs were 0.7838 (95% CI, 0.6709–0.8967; *p* < 0.0001) and 0.8184 (95% CI, 0.6508–0.9859; *p* = 0.0006), respectively (Supplementary Figure S4 and Supplementary Table S7). Thus, the discriminatory performance of both genes remained evident after the landmark restriction, with stronger discrimination generally observed at week 24.

Overall, the landmark analyses supported the findings of the primary ROC analyses. The discriminatory ability of IFI44L and SAMHD1 remained evident after patients who had already achieved the corresponding treatment endpoint at the time of biomarker assessment were excluded. The detailed results are presented in Supplementary Table S7, and the corresponding ROC curves are shown in Supplementary Figure S4.

## Discussion

Pegylated interferon-alpha (Peg-IFN-α) continues to occupy an important place in the treatment of chronic hepatitis B (CHB), particularly for patients in whom a finite course of therapy and the possibility of achieving functional cure are clinically relevant considerations. Contemporary treatment guidance continues to include Peg-IFN-α among the available therapeutic options for appropriately selected patients (European Association for the Study of the Liver, 2025; You et al., 2023). Its value extends beyond direct suppression of HBV replication, as the drug can engage host interferon-dependent pathways and promote a sustained antiviral state that may persist after treatment has been completed. Consistent with this therapeutic rationale, HBsAg loss rates exceeding 30% have been reported in selected patient populations receiving Peg-IFN-α-containing regimens (Chu et al., 2022; Li et al., 2024b). The benefit, however, is far from uniform. A substantial proportion of patients do not achieve HBsAg clearance despite completing a standard treatment course, emphasizing the marked interindividual variability in interferon responsiveness among patients with CHB. This heterogeneity remains an important limitation of Peg-IFN-α therapy because treatment efficacy cannot be reliably inferred from the treatment regimen alone. Biomarkers that identify, early during therapy, patients with a higher likelihood of achieving a meaningful response could therefore improve treatment selection and help limit unnecessary exposure to an ineffective course of interferon-based therapy. The heterogeneous clinical response to Peg-IFN-α is unlikely to be explained solely by differences in circulating viral burden. Its antiviral activity involves coordinated induction of interferon-stimulated genes (ISGs) and broader changes in host antiviral and immune signaling networks (Schoggins et al., 2011; Li et al., 2022). PBMCs therefore represent a readily accessible peripheral compartment in which treatment-related alterations in host immune transcription can be monitored. Previous work has shown that host RNA expression profiles in peripheral blood can capture clinically relevant features of antiviral immune responses in infectious diseases, including HBV infection (Gliddon et al., 2018). Lu et al. reported that PBMC-derived interferon gamma-inducible protein 16 (IFI16) participates in sensing HBV-associated signals and may influence antiviral immune responses in CHB (Lu et al., 2021). Han et al. similarly found that treatment-related activation of ISGs, including STAT1 and MX, in PBMCs was associated with antiviral response and with the extent of HBsAg decline from baseline to week 48 (Han et al., 2017). Together, these observations provide a biological and clinical rationale for examining dynamic peripheral transcriptional responses as potential markers of treatment sensitivity. Nevertheless, the clinical meaning of individual ISGs during Peg-IFN-α therapy remains incompletely established. In particular, it has remained uncertain whether longitudinal changes in individual ISGs provide information beyond evidence of interferon exposure itself and whether such changes are linked to virological or serological outcomes.

Our findings address this issue by focusing on IFI44L and SAMHD1 in PBMCs collected from HBeAg-negative patients receiving Peg-IFN-α. Both genes are established interferon-responsive components of the host antiviral response and have previously been associated with antiviral defense against HBV (Du et al., 2024; Schoggins et al., 2011; Nosaka et al., 2022; Sommer et al., 2016; Chen et al., 2014). At baseline, their expression was lower in untreated patients with HBeAg-negative CHB than in healthy controls. This pattern is consistent with reduced basal expression of these interferon-responsive transcripts in circulating immune cells during chronic HBV infection. More importantly, however, the on-treatment expression of these genes differed according to the treatment outcome achieved. Their expression increased significantly from baseline at weeks 12 and 24 in patients who achieved VR or SR, whereas no significant longitudinal changes were observed in the corresponding non-responder groups. The more informative feature was therefore not baseline expression itself, but the longitudinal induction of these transcripts after treatment initiation. This finding is clinically relevant because exposure to the same interferon-based regimen did not produce an equivalent transcriptional response across patients. The observed differences are consistent with the possibility that interindividual variation in the magnitude of the on-treatment interferon-responsive transcriptional response is associated with differences in treatment response. At the same time, the present study does not establish why some patients exhibit a stronger induction of IFI44L or SAMHD1 than others, nor does it demonstrate that either gene directly controls the treatment outcome. Their longitudinal expression should therefore be viewed as a marker of treatment-associated host responsiveness rather than as evidence of a causal mechanism. This distinction is important when interpreting their potential biomarker value. Unlike a single pretreatment measurement, a dynamic marker may capture the host response that emerges after interferon exposure and may therefore provide information that is not evident from baseline transcript levels alone. In this context, treatment-associated trajectories of IFI44L and SAMHD1 may help distinguish patients with more favorable virological or serological outcomes at week 48.

HBsAg loss is a central serological milestone toward functional cure in CHB and remains an important measure of treatment success (Terrault et al., 2018; Wu et al., 2025). In our cohort, 41 of 76 patients treated with Peg-IFN-α achieved VR by week 48, corresponding to a rate of 53.95%, while 28 patients achieved SR, giving an SR rate of 36.84%. The observed response rates were broadly comparable to those reported in selected HBeAg-negative populations receiving Peg-IFN-α-based treatment, although differences in baseline virological status and prior antiviral exposure limit direct comparison (Huang et al., 2017). The longitudinal behavior of HBsAg and HBV DNA further illustrates that virological suppression and HBsAg clearance, although closely related, represent distinct stages of treatment response. During treatment, serum HBsAg declined progressively, yet the proportion of patients who achieved complete HBsAg loss remained appreciably smaller than the proportion showing a measurable decrease in circulating HBsAg. This difference is biologically plausible because serum HBsAg is influenced, at least in part, by intrahepatic HBV transcriptional activity and the cccDNA reservoir, as well as by HBsAg-producing transcripts derived from integrated HBV DNA, and therefore may not fall in parallel with the more rapid decline in circulating HBV DNA after antiviral treatment begins (Gao et al., 2017; Chan et al., 2007; Meier et al., 2021). HBV DNA suppression may consequently precede the deeper virological transition represented by HBsAg loss. These distinct kinetics provide a rationale for evaluating both markers when characterizing the response to Peg-IFN-α rather than treating HBsAg decline and HBV DNA suppression as interchangeable measures. The temporal behavior of HBsAg in our cohort also helps place the observed treatment responses in clinical context. Reduction in serum HBsAg occurred progressively over the treatment period, whereas complete clearance and anti-HBs seroconversion accumulated more gradually. In this study, HBsAg loss was therefore evaluated as the primary serological endpoint, whereas anti-HBs seroconversion was considered a subsequent and separately reported serological event. A reduction in HBsAg concentration, although clinically informative, should therefore not be equated with functional cure. Importantly, the week-48 HBsAg-loss endpoint used to define SR in this study should not be interpreted as functional cure, because post-treatment follow-up was not performed to establish sustained HBsAg loss and HBV DNA suppression. The progression from reduced HBsAg burden to HBsAg loss and subsequent anti-HBs seroconversion represents a more advanced serological response and is achieved by only a subset of treated patients. The different kinetics of HBsAg and HBV DNA therefore support their use as complementary rather than interchangeable indicators of treatment response. Another observation requiring consideration was the higher ALT and AST levels detected at week 24 among patients who achieved VR. The temporal context is noteworthy: these biochemical differences emerged at a time when the VR group also showed lower levels of HBV DNA and stronger changes in interferon-responsive gene expression. One possible explanation is that these aminotransferase elevations accompanied a period of heightened host immune activity during effective treatment, as transient biochemical flares have been described during interferon-based therapy and may reflect immune-mediated injury to HBV-infected hepatocytes (Chang and Liaw, 2014). Nevertheless, the present study was not designed to determine the cellular or molecular origin of the week 24 ALT and AST elevations. Accordingly, the observed biochemical changes should be interpreted as treatment-associated findings that coincided with virological and transcriptional differences, rather than as direct evidence of an immune-mediated mechanism. Clarifying whether these transient aminotransferase elevations reflect enhanced antiviral immunity, hepatocyte turnover, or other treatment-related processes will require studies incorporating direct measures of intrahepatic immune activity.

The relationship between conventional clinical markers and treatment outcome was also evident in the longitudinal comparison of the response groups. Patients who ultimately achieved VR or SR generally had lower HBsAg and HBV DNA levels during the pretreatment and early-treatment assessments, and the multivariable analyses showed that HBV DNA remained independently associated with VR, whereas HBsAg retained an independent association with SR. These observations are consistent with the recognized importance of viral burden and HBsAg kinetics in response to interferon-based treatment, although previous studies have not identified a uniformly consistent set of conventional baseline factors across different patient populations (Sonneveld et al., 2012; Sonneveld et al., 2013; Rijckborst et al., 2010; Brunetto et al., 2009). This variability across studies suggests that conventional virological and biochemical measures, while clinically useful, may not fully capture the biological heterogeneity that determines interferon responsiveness. Our findings therefore support a complementary framework in which conventional markers of viral burden are interpreted alongside dynamic measures of host interferon responsiveness. The clinical variables identify aspects of the pretreatment and early-treatment viral state, whereas longitudinal IFI44L and SAMHD1 expression reflect the transcriptional response that develops after interferon exposure. Although HBsAg and HBV DNA are both virological markers of HBV infection, they are not necessarily interchangeable, particularly in HBeAg-negative CHB. Their relationship varies across clinical phases, and the two markers may provide complementary information on viral burden and antigen production. The absence of substantial multicollinearity indicated that their concurrent inclusion did not introduce problematic redundancy among the predictors. The fact that the expression of both genes increased preferentially in patients who later achieved VR or SR, remained independently associated with the corresponding outcomes in multivariable analyses, and correlated with reductions in HBsAg and HBV DNA further strengthens their potential relevance as response-associated biomarkers. Importantly, these relationships are associative rather than causal. The present data support the clinical relevance of monitoring dynamic host responses during Peg-IFN-α treatment, but they do not establish that IFI44L or SAMHD1 directly mediates HBV clearance. Rather, these transcripts may serve as measurable indicators of an interferon-responsive host state that accompanies more favorable virological and serological trajectories.

Interferon-stimulated genes (ISGs) are central components of the host response to viral infection, and their involvement in antiviral defense against HBV has been investigated extensively (Allweiss et al., 2014; Fletcher et al., 2015). Even so, treatment with Peg-IFN-α produces markedly different outcomes across individuals with CHB, and reliable markers that can identify patients more likely to benefit from interferon-based therapy remain needed. In our cohort, the greater decline in serum HBsAg among patients who ultimately achieved treatment response provided a clinically relevant framework for examining whether treatment-induced changes in interferon-responsive transcription were associated with outcome. The longitudinal behavior of IFI44L and SAMHD1 was not characterized by a uniform increase in all treated patients. Rather, their expression changed in relation to the eventual response status. Among patients who achieved VR, both transcripts increased significantly during therapy, and the separation from the NVR group was particularly evident by week 24. The same overall pattern was observed when SR was used to define treatment success, with higher IFI44L and SAMHD1 expression in the SR group than in the NSR group at both weeks 12 and 24. The concordant findings for two clinically distinct treatment endpoints further support the association between greater on-treatment induction of these genes and more favorable responses to Peg-IFN-α. However, the nature of this relationship should be interpreted within the limits of the observational study design. Our data show that higher expression of IFI44L and SAMHD1 accompanies better treatment outcomes, but they do not demonstrate that induction of either gene directly causes improved HBV control. At the present stage, these ISGs are therefore better regarded as candidate indicators of treatment-associated host responsiveness than as established mechanistic determinants of therapeutic efficacy. This interpretation is also consistent with the longitudinal analyses showing that on-treatment expression, rather than baseline transcript abundance alone, carried the strongest clinical information.

The correlation analyses provided additional support for the relationship between the treatment-related changes in IFI44L and SAMHD1 and the decline in circulating viral markers. At both weeks 12 and 24, higher expression of either gene was associated with a greater reduction in HBsAg and HBV DNA. The reproducibility of these relationships across two treatment assessments is relevant because it makes it less likely that the observed associations simply reflected a transient fluctuation at a single sampling point. More importantly, the analyses linking week 12 expression with the subsequent changes measured at week 24 introduced a temporal component that was not captured by the contemporaneous correlations alone. IFI44L and SAMHD1 levels at week 12 were each significantly associated with the subsequent decline in HBsAg and HBV DNA at week 24. From a translational standpoint, this temporal relationship is of particular interest because it suggests that an early treatment-associated transcriptional response may contain information related to subsequent virological change. The findings are also consistent with the possibility that interindividual differences in early interferon responsiveness influence the extent to which effective antiviral control is established during continued treatment. Nevertheless, the present data cannot distinguish between two possibilities because stronger induction of IFI44L or SAMHD1 may either contribute to improved viral control or simply mark a broader host state that is more responsive to interferon and therefore more likely to achieve successful treatment. The current results should consequently be interpreted as evidence supporting biomarker relevance rather than as evidence for a direct antiviral role of either gene in this clinical setting. Their detection in PBMCs further supports their potential translational utility by providing an accessible and minimally invasive readout of treatment-associated host responsiveness. With independent validation, these longitudinal profiles may help characterize differences in treatment-associated host responses and their relationship to week-48 treatment outcomes.

The potential relevance of IFI44L and SAMHD1 is not limited to antiviral immunity. Both genes have also been studied in liver disorders beyond chronic HBV infection, including hepatocellular carcinoma (HCC). Lower IFI44L expression has been associated with tumor recurrence and poorer survival among patients with HCC (Huang et al., 2018). SAMHD1 has likewise been linked to tumor-suppressive activity in HCC and has been reported in relation to the development of metabolic dysfunction-associated steatotic liver disease (Shao et al., 2025; Yin et al., 2026). These observations indicate that both genes have broader biological relevance in liver disease, although these findings arise largely from tissue-based or experimental studies and do not directly establish their relevance to treatment response in CHB. Whether dynamic changes in these two ISGs have clinical relevance for treatment response in HBeAg-negative CHB had not, however, been established. In the present cohort, treatment-associated expression of IFI44L and SAMHD1 remained significantly associated with the eventual response in both univariate and multivariable analyses. Conventional virological variables also retained prognostic information, with HBsAg and HBV DNA showing significant associations in the corresponding analyses, in line with previous reports (Zhang et al., 2020; Wu et al., 2020). The coexistence of host-derived and viral associations suggests that these measures may capture complementary dimensions of treatment responsiveness rather than interchangeable manifestations of a single biological process.

Our ROC analyses further addressed whether the expression of IFI44L and SAMHD1 could distinguish patients according to the treatment outcome ultimately reached at week 48. Both genes showed a clear temporal improvement in apparent discriminatory performance within the study cohort. Before treatment, their AUC values were close to 0.50, indicating little ability to separate eventual responders from non-responders. Discrimination improved substantially at week 12 and reached its highest level at week 24 for both VR and SR. This temporal pattern is notable because the strongest discrimination emerged only after Peg-IFN-α exposure, rather than at baseline. If pretreatment differences in constitutive ISG expression were sufficient to determine treatment outcome, baseline measurements might be expected to show a stronger separation. Instead, the improvement after treatment initiation suggests that the host response elicited by interferon may provide information that is not apparent from baseline transcript abundance alone. In this respect, the on-treatment expression of IFI44L and SAMHD1 may provide more clinically informative discrimination than their pretreatment levels, potentially reflecting the host interferon-response behavior that emerges after therapeutic stimulation. The findings therefore support the potential utility of these transcripts as candidate dynamic on-treatment biomarkers rather than as static baseline predictors. This distinction also has implications for the possible clinical use of these markers. HBsAg and HBV DNA remain essential components of treatment assessment and provide important information on the virological state. They do not, however, directly describe how effectively an individual host responds to exogenous interferon. IFI44L and SAMHD1 could therefore serve as complementary measures of the host component of treatment response rather than substitutes for established viral biomarkers. A future strategy that integrates dynamic host-response measurements with conventional HBV markers may provide a more comprehensive assessment of treatment-associated host-virus interactions during Peg-IFN-α therapy. Such an approach might ultimately help stratify patients according to their likelihood of achieving a favorable week-48 treatment outcome during Peg-IFN-α therapy, although its value for treatment decision-making remains to be established. However, this clinical application remains hypothetical. Demonstrating incremental value over established on-treatment markers such as HBsAg and HBV DNA will require prospective validation in independent cohorts and assessment of whether these additional measurements improve treatment decisions. The landmark analyses further supported the interpretation of the on-treatment ROC findings. After accounting for patients who had already achieved the corresponding treatment status at the time of biomarker assessment, the discriminatory performance of IFI44L and SAMHD1 remained evident. These findings suggest that the observed association between on-treatment expression of these genes and treatment outcome was not limited to patients who had already responded before biomarker measurement. The landmark results therefore strengthen the potential relevance of IFI44L and SAMHD1 as dynamic markers of host interferon responsiveness during Peg-IFN-α therapy.

Several limitations should be considered when interpreting these findings. First, the observation period was limited to the 48-week treatment course and did not include systematic post-treatment follow-up. Therefore, the durability of HBsAg loss, the persistence of virological or serological responses after treatment discontinuation, and the achievement of a sustained functional cure could not be determined. Second, HBV genotype information was unavailable for all participants and consequently could not be incorporated into the multivariable analyses. Previous studies, particularly in HBeAg-positive CHB, have suggested that genotype B may be associated with a more favorable interferon response than genotype C (Wai et al., 2002; Yan et al., 2014). Whether a similar relationship exists in HBeAg-negative patients, and whether HBV genotype modifies the association between IFI44L or SAMHD1 expression and treatment response, remains uncertain. Future studies incorporating complete genotype information may therefore determine whether the relationship between IFI44L or SAMHD1 expression and treatment response varies across viral genotypes. Third, the cohort consisted exclusively of HBeAg-negative patients with moderate-to-high baseline viral loads. This clinical profile may limit extrapolation of the findings to patients with lower HBV DNA levels and may have introduced a degree of selection bias. Validation in populations covering a wider range of baseline viremia will be necessary to establish whether the discriminatory performance of these markers is maintained in patients with low viral loads. Fourth, the composition of circulating PBMCs may change during Peg-IFN-α treatment and could have contributed to the observed changes in IFI44L and SAMHD1 expression. PBMCs are practical and minimally invasive to collect, but they do not reproduce the cellular environment of the liver, which remains the principal site of HBV replication. Future work using cell subset-specific measurements, single-cell transcriptomic approaches, and liver tissue analyses will therefore be important for identifying the cell populations responsible for the observed transcriptional patterns and for determining how closely peripheral ISG dynamics reflect antiviral events within the liver. Finally, this investigation was conducted at a single center in a relatively small cohort consisting exclusively of HBeAg-negative CHB patients. Larger, multicenter studies involving broader clinical populations will be needed to independently reproduce the present observations and establish the extent to which they can be generalized across the wider CHB population.

## Conclusion

In summary, the present study identifies a treatment-associated pattern of IFI44L and SAMHD1 expression in PBMCs of HBeAg-negative patients with CHB receiving Peg-IFN-α. Greater induction of both transcripts was associated with more favorable virological and serological outcomes, as well as greater declines in HBsAg and HBV DNA. Their discriminatory performance increased after treatment initiation and was highest at week 24, supporting their potential as candidate dynamic markers of host interferon responsiveness during Peg-IFN-α therapy.

## Data Availability

The raw data supporting the conclusions of this article will be made available by the authors, without undue reservation.
