# Supplementary material for: Dynamic changes in IFI44L and SAMHD1 mRNA levels in PBMCs are associated with treatment response in HBeAg-negative chronic hepatitis B during peg-IFN-α therapy
Source: Front Microbiol. 2026 Sep 25;17:1976096. doi: 10.3389/fmicb.2026.1976096 (PMC13649976; doi:10.3389/fmicb.2026.1976096)
Supplement: Supplementary file 1 [file Table_1.DOCX]

| **Table S1** Primer sequences used in this study | |
| --- | --- |
| Primers sequence(5’-3’) | |
| GAPDH RP | 5’-TGACACGTTGGCAGTGG-3’ |
| GAPDH FP | 5’-GGGGCTCTCCAGAACATC-3’ |
| IFI44L RP | 5’-GATGAACTTTGACATCCCTGAG-3’ |
| IFI44L FP | 5’-GATTGCAGTGAGGTTCTTC-3’ |
| SAMHD1 RP | 5’- CAACTGTGGATCCGTGGAGTATA-3’ |
| SAMHD1 FP | 5’-TCGGCAACCTCATCGATATAATGA-3’ |
