# Supplementary material for: Dynamic changes in IFI44L and SAMHD1 mRNA levels in PBMCs are associated with treatment response in HBeAg-negative chronic hepatitis B during peg-IFN-α therapy
Source: Front Microbiol. 2026 Sep 25;17:1976096. doi: 10.3389/fmicb.2026.1976096 (PMC13649976; doi:10.3389/fmicb.2026.1976096)
Supplement: Supplementary file 2 [file Table_2.DOCX]

| **Table S2** Treatment response during Peg-IFN-α treatment | | | | |
| --- | --- | --- | --- | --- |
| Treatment response (n, %) | 12w | 24w | 36w | 48w |
| HBV DNA < 50 10 IU/ml | 11(14.47%) | 26(34.21%) | 35(46.05%) | 41(53.95%) |
| HBsAg clearance | 5(6.58%) | 12(15.79%) | 18(23.68%) | 28(36.84%) |
| HBsAg seroconversion | 0(0.00%) | 4(5.26%) | 8(10.53%) | 20(26.32%) |
