# Supplementary material for: Dynamic changes in IFI44L and SAMHD1 mRNA levels in PBMCs are associated with treatment response in HBeAg-negative chronic hepatitis B during peg-IFN-α therapy
Source: Front Microbiol. 2026 Sep 25;17:1976096. doi: 10.3389/fmicb.2026.1976096 (PMC13649976; doi:10.3389/fmicb.2026.1976096)
Supplement: Supplementary file 3 [file Table_3.DOCX]

| **Table S3** On-treatment variables associated with virological response according to univariate and multivariate analyses | | | | | | | | | | | | |
| --- | --- | --- | --- | --- | --- | --- | --- | --- | --- | --- | --- | --- |
| Variables | Univariate analyses |  | Multivariate analyses |  | Univariate analyses |  | Multivariate analyses |  | Univariate analyses |  | Multivariate analyses |  |
|  | OR(95%CI) | P-value | aOR(95%CI) | P-value | OR(95%CI) | P- value | aOR(95%CI) | P-value | OR(95%CI) | P-value | aOR(95%CI) | P-  value |
|  | **Week0** |  |  |  | **Week12** |  |  |  | **Week24** |  |  |  |
| Gender | 0.610(0.243, 1.530) | 0.2927 |  |  | 0.610(0.243,1.530) | 0.2927 |  |  | 0.610(0.243,1.530) | 0.2927 |  |  |
| Age | 1.005(0.963, 1.048) | 0.8036 |  |  | 1.005(0.963,1.048) | 0.8036 |  |  | 1.005(0.963,1.048) | 0.8036 |  |  |
| HBsAg | 0.142(0.053, 0.377) | **0.0008** | 0.170(0.063,1.457) | 0.0552 | 0.201(0.089,0.452) | **0.0001** | 0.339(0.098,  1.164) | 0.0813 | 0.225(0.112  ,0.453) | **0.0002** | 0.426(0.096  ,1.880) | 0.2602 |
| HBV DNA | 0.110(0.032, 0.373) | **0.0004** | 0.094(0.018,0.479) | **0.0043** | 0.119(0.043,0.332) | **0.0042** | 0.047(0.006,  0.323) | **0.0018** | 0.135(0.052,0.347) | **0.0051** | 0.097(0.018  ,0.502) | **0.0054** |
| ALT | 1.005(0.994, 1.017) | 0.9455 |  |  | 1.004(0.996,0.996) | 0.3214 |  |  | 1.022(1.001,1.043) | **0.0387** | 0.988(0.948  ,1.030) | 0.5954 |
| AST | 1.001(0.992, 1.007) | 0.9785 |  |  | 1.006(0.996,1.016) | 0.1780 |  |  | 1.034(1.006,1.063) | **0.0163** | 1.105(0.973  ,1.358) | 0.6541 |
| WBC | 1.174(0.891, 1.545) | 0.2530 |  |  | 0.812(0.602,1.094) | 0.1710 |  |  | 0.893(0.698,1.144) | 0.3721 |  |  |
| PLT | 1.007(0.999, 1.015) | 0.0519 |  |  | 1.001(0.994,1.008) | 0.7559 |  |  | 0.997(0.989,1.003) | 0.2884 |  |  |

**Continued Table S3** On-treatment variables associated with virological response according to univariate and multivariate analyses

| Variables | Univariate analyses |  | Multivariate analyses |  | Univariate analyses |  | Multivariate analyses |  | Univariate analyses |  | Multivariate analyses |  |
| --- | --- | --- | --- | --- | --- | --- | --- | --- | --- | --- | --- | --- |
|  | OR(95%CI) | P-value | aOR(95%CI) | P-value | OR(95%CI) | P-value | aOR(95%CI) | P-value | OR(95%CI) | P-value | aOR(95%CI) | P-  value |
| IFI44L | 0.762(0.492,1.181) | 0.2246 |  |  | 1.321(1.050,1.662) | **0.0174** | 1.578(1.047,  2.378) | **0.0289** | 1.805(1.348,2.415) | **0.0012** | 2.267(1.352  ,3.802) | **0.0019** |
| SAMHD1 | 0.960(0.6707,1.3745) | 0.8242 |  |  | 2.219(1.435,3.431) | **0.0003** | 1.746(1.074,  2.836) | **0.0243** | 1.904(1.399,2.590) | **0.0001** | 2.563(1.274  ,5.156) | **0.0083** |
| Values expressed as odds ratio (OR) and 95% confidence interval (CI). aOR, adjusted odds ratio; IFI44L, Interferon-induced protein 44-like; SAMHD1, Sterile alpha motif and histidine–aspartic acid domain containing protein 1; HBsAg, hepatitis B surface antigen; ALT, alanine aminotransferase; AST, aspartate aminotransferase; WBC, white blood cells; PLT: platelet. Bold values are statistically significant P < 0.05. | | | | | | | | | | | | |
