# Supplementary material for: Dynamic changes in IFI44L and SAMHD1 mRNA levels in PBMCs are associated with treatment response in HBeAg-negative chronic hepatitis B during peg-IFN-α therapy
Source: Front Microbiol. 2026 Sep 25;17:1976096. doi: 10.3389/fmicb.2026.1976096 (PMC13649976; doi:10.3389/fmicb.2026.1976096)
Supplement: Supplementary file 4 [file Table_4.DOCX]

| **Table S4** On-treatment variables associated with serological response according to univariate and multivariate analyses. | | | | | | | | | | | | |
| --- | --- | --- | --- | --- | --- | --- | --- | --- | --- | --- | --- | --- |
| Variables | Univariate analyses |  | Multivariate analyses |  | Univariate analyses |  | Multivariate analyses |  | Univariate analyses |  | Multivariate analyses |  |
|  | OR(95%CI) | P-  value | aOR(95%CI) | P-  value | OR(95%CI) | P-  value | aOR(95%CI) | P-value | OR(95%CI) | P-value | aOR(95%CI) | P-  value |
|  | **Week0** |  |  |  | **Week12** |  |  |  | **Week24** |  |  |  |
| Gender | 0.831(0.322  ,2.148) | 0.7039 |  |  | 0.831(0.322,2.148) | 0.7039 |  |  | 0.831(0.322,2.148) | 0.7039 |  |  |
| Age | 0.831(0.322  ,2.148) | 0.7038 |  |  | 0.831(0.322,2.148) | 0.7038 |  |  | 0.831(0.322,2.148) | 0.7038 |  |  |
| HBsAg | 0.054(0.015  ,0.190) | **0.0005** | 0.064(0.017  ,0.232) | **0.0002** | 0.160(0.069,0.371) | **0.0019** | 0.168(0.068  ,0.417) | **0.0001** | 0.176(0.076,0.405) | **0.0044** | 0.137(0.033  ,0.561) | **0.0056** |
| HBV DNA | 0.186(0.058  ,0.597) | **0.0046** | 0.245(0.041  ,1.441) | 0.1200 | 0.413(0.249,0.685) | **0.0006** | 0.826(0.395  ,1.727) | 0.6126 | 0.343(0.213,0.552) | **0.0001** | 0.855(0.407  ,1.798) | 0.6812 |
| ALT | 1.002(0.991  ,1.014) | 0.6508 |  |  | 1.003(0.995,1.011) | 0.3805 |  |  | 1.010(0.993,1.027) | 0.2353 |  |  |
| AST | 1.002(0.994  ,1.010) | 0.5344 |  |  | 1.006(0.997,1.016) | 0.1683 |  |  | 1.003(0.988,1.019) | 0.6911 |  |  |
| WBC | 0.899(0.678  ,1.193) | 0.4636 |  |  | 0.784(0.565,1.088) | 0.1460 |  |  | 0.890(0.684,1.159) | 0.3896 |  |  |
| PLT | 0.999(0.992  ,1.007) | 0.9071 |  |  | 1.001(0.992,1.007) | 0.9688 |  |  | 0.985(0.902,1.004) | 0.1560 |  |  |

**Continued Table S4** On-treatment variables associated with serological response according to univariate and multivariate analyses.

| Variables | Univariate analyses |  | Multivariate analyses |  | Univariate analyses |  | Multivariate analyses |  | Univariate analyses |  | Multivariate analyses |  |
| --- | --- | --- | --- | --- | --- | --- | --- | --- | --- | --- | --- | --- |
|  | OR(95%CI) | P-value | aOR(95%CI) | P-value | OR(95%CI) | P-  value | aOR(95%CI) | P-value | OR(95%CI) | P-value | aOR(95%CI) | P-  value |
| IFI44L | 0.769(0.524  ,1.129) | 0.0755 |  |  | 1.421(1.130  ,1.788) | **0.0026** | 1.443(1.114  ,1.868) | **0.0053** | 1.458(1.208,1.761) | **0.0086** | 1.616(1.150  ,2.270) | **0.0055** |
| SAMHD1 | 0.894(0.619  ,1.293) | 0.5541 |  |  | 1.775(1.302  ,2.420) | **0.0002** | 1.574(1.055  ,2.349) | **0.0262** | 1.734(1.408, 2.136) | **0.0001** | 2.162(1.306  ,3.579) | **0.0026** |
| Values expressed as odds ratio (OR) and 95% confidence interval (CI). aOR, adjusted odds ratio; IFI44L, Interferon-induced protein 44-like; SAMHD1, Sterile alpha motif and histidine–aspartic acid domain containing protein 1; HBsAg, hepatitis B surface antigen; ALT, alanine aminotransferase; AST, aspartate aminotransferase; WBC, white blood cells; PLT: platelet. Bold values are statistically significant P < 0.05. | | | | | | | | | | | | |
