# Supplementary material for: Dynamic changes in IFI44L and SAMHD1 mRNA levels in PBMCs are associated with treatment response in HBeAg-negative chronic hepatitis B during peg-IFN-α therapy
Source: Front Microbiol. 2026 Sep 25;17:1976096. doi: 10.3389/fmicb.2026.1976096 (PMC13649976; doi:10.3389/fmicb.2026.1976096)
Supplement: Supplementary file 5 [file Table_5.DOCX]

| **Table S5** Collinearity diagnostics for continuous covariates included in the multivariable logistic regression models. | | | | |
| --- | --- | --- | --- | --- |
| **Outcome** | **Time point** | **Variable** | **Tolerance** | **VIF** |
| **Virological Response (VR)** | Week 0 | HBsAg | 0.6173 | 1.62 |
|  |  | HBV DNA | 0.6173 | 1.62 |
|  | Week 12 | HBsAg | 0.5376 | 1.86 |
|  |  | HBV DNA | 0.5051 | 1.98 |
|  |  | IFI44L | 0.5848 | 1.71 |
|  |  | SAMHD1 | 0.5682 | 1.76 |
|  | Week 24 | HBsAg | 0.5236 | 1.91 |
|  |  | HBV DNA | 0.4878 | 2.05 |
|  |  | ALT | 0.4464 | 2.24 |
|  |  | AST | 0.4587 | 2.18 |
|  |  | IFI44L | 0.5952 | 1.68 |
|  |  | SAMHD1 | 0.5747 | 1.74 |
| **Serological Response (SR)** | Week 0 | HBsAg | 0.6173 | 1.62 |
|  |  | HBV DNA | 0.6173 | 1.62 |
|  | Week 12 | HBsAg | 0.5376 | 1.86 |
|  |  | HBV DNA | 0.5051 | 1.98 |
|  |  | IFI44L | 0.5848 | 1.71 |
|  |  | SAMHD1 | 0.5682 | 1.76 |
|  | Week 24 | HBsAg | 0.5464 | 1.83 |
|  |  | HBV DNA | 0.5319 | 1.88 |
|  |  | IFI44L | 0.6061 | 1.65 |
|  |  | SAMHD1 | 0.5848 | 1.71 |
| VIF, variance inflation factor; VR, virological response; SR, serological response; HBsAg, hepatitis B surface antigen; HBV DNA, hepatitis B virus DNA; ALT, alanine aminotransferase; AST, aspartate aminotransferase; IFI44L, interferon-induced protein 44-like; SAMHD1, sterile alpha motif and histidine–aspartic acid domain-containing protein 1. Tolerance was calculated as the reciprocal of the corresponding VIF. Collinearity diagnostics were performed using the same predictor sets as those used in the corresponding multivariable logistic regression models. | | | | |
