# Supplementary material for: Dynamic changes in IFI44L and SAMHD1 mRNA levels in PBMCs are associated with treatment response in HBeAg-negative chronic hepatitis B during peg-IFN-α therapy
Source: Front Microbiol. 2026 Sep 25;17:1976096. doi: 10.3389/fmicb.2026.1976096 (PMC13649976; doi:10.3389/fmicb.2026.1976096)
Supplement: Supplementary file 6 [file Table_6.DOCX]

| **Table S6** The predictive performance of IFI44L and SAMHD1 mRNA levels in predicting VR and SR to 48 weeks of Peg-IFN-α treatment. | | | | | | | | | | |
| --- | --- | --- | --- | --- | --- | --- | --- | --- | --- | --- |
|  |  | IFI44L | SAMHD1 |  | IFI44L | SAMHD1 |  | IFI44L | SAMHD1 |  |
| VR prediction |  |  | Week 0 |  |  | Week12 |  |  | Week24 |  |
|  | AUC | 0.5268 | 0.5038 |  | 0.6627 | 0.8272 |  | 0.8822 | 0.8627 |  |
|  | (95% CI) | (0.3914 - 0.6623) | (0.3696 -  0.6381) |  | (0.5408 -   0.7846) | (0.7319 - 0.9225) |  | (0.8089 -0.9556) | (0.7740 - 0.9514) |  |
|  | Cut-off value | 2.4951 | 1.6108 |  | 3.4441 | 1.7899 |  | 3.7326 | 5.3252 |  |
|  | Sensitivity (%) | 92.70 | 53.70 |  | 66.60 | 78.00 |  | 75.30 | 75.60 |  |
|  | Specificity (%) | 25.70 | 65.70 |  | 94.30 | 80.00 |  | 91.40 | 92.10 |  |
|  | P value | 0.6883 | 0.9543 |  | **0.0150** | **< 0.0001** |  | **< 0.0001** | **< 0.0001** |  |
| SR prediction |  |  | Week 0 |  |  | Week12 |  |  | Week24 |  |
|  | AUC | 0.5045 | 0.5074 |  | 0.8021 | 0.7790 |  | 0.8445 | 0.8400 |  |
|  | (95% CI) | (0.3759 - 0.6330) | (0.3727 -  0.6421) |  | (0.7060 -  0.8982) | (0.6740 - 0.8840) |  | (0.7583 -  0.9307) | (0.7444 - 0.9357) |  |
|  | Cut-off value | 2.0861 | 3.1086 |  | 0.6946 | 1.6020 |  | 1.6029 | 2.8310 |  |
|  | Sensitivity (%) | 85.70 | 56.60 |  | 78.90 | 85.70 |  | 85.40 | 82.10 |  |
|  | Specificity (%) | 35.40 | 27.10 |  | 65.30 | 60.40 |  | 82.50 | 81.20 |  |
|  | P value | 0.9485 | 0.9142 |  | **< 0.0001** | **0.0001** |  | **< 0.0001** | **< 0.0001** |  |
| IFI44L, Interferon-induced protein 44-like; SAMHD1, Sterile alpha motif and histidine-aspartic acid domain containing protein 1; AUC, area under ROC curve; CI, confidence interval; VR, virological response; SR, serological response; Bold values are statistically significant P < 0.05. | | | | | | | | | | |
