# Supplementary material for: Dynamic changes in IFI44L and SAMHD1 mRNA levels in PBMCs are associated with treatment response in HBeAg-negative chronic hepatitis B during peg-IFN-α therapy
Source: Front Microbiol. 2026 Sep 25;17:1976096. doi: 10.3389/fmicb.2026.1976096 (PMC13649976; doi:10.3389/fmicb.2026.1976096)
Supplement: Supplementary file 7 [file Table_7.DOCX]

| **Table S7** Landmark analysis of IFI44L and SAMHD1 for treatment response during Peg-IFN-α therapy. | | | | | | | | | | |
| --- | --- | --- | --- | --- | --- | --- | --- | --- | --- | --- |
|  |  |  |  |  | IFI44L | SAMHD1 |  | IFI44L | SAMHD1 |  |
| VR prediction |  |  |  |  |  | Week12 |  |  | Week24 |  |
|  | AUC |  |  |  | 0.6419 | 0.7838 |  | 0.7939 | 0.8184 |  |
|  | (95% CI) |  |  |  | (0.5055 - 0.7783) | (0.6709 - 0.8967) |  | (0.6613 - 0.9264) | (0.6508 - 0.9859) |  |
|  | Cut-off value |  |  |  | 0.7128 | 0.7873 |  | 1.5886 | 3.7748 |  |
|  | Sensitivity (%) |  |  |  | 63.00 | 59.30 |  | 75.00 | 71.40 |  |
|  | Specificity (%) |  |  |  | 96.50 | 96.50 |  | 94.20 | 95.50 |  |
|  | P value |  |  |  | **0.0499** | **< 0.0001** |  | **0.0014** | **0.0006** |  |
| SR prediction |  |  |  |  |  | Week12 |  |  | Week24 |  |
|  | AUC |  |  |  | 0.7899 | 0.7319 |  | 0.8110 | 0.8065 |  |
|  | (95% CI) |  |  |  | (0.6873 - 0.8924) | (0.6131 - 0.8506) |  | (0.7064 - 0.9156) | (0.6765 - 0.9366) |  |
|  | Cut-off value |  |  |  | 0.6235 | 1.4945 |  | 2.0149 | 2.4471 |  |
|  | Sensitivity (%) |  |  |  | 78.90 | 87.00 |  | 93.40 | 85.70 |  |
|  | Specificity (%) |  |  |  | 58.30 | 66.20 |  | 64.60 | 84.10 |  |
|  | P value |  |  |  | **< 0.0001** | **0.0017** |  | **0.0004** | **0.0005** |  |
| For the landmark analyses, patients who had already achieved the corresponding treatment response at the specified landmark time point were excluded. IFI44L, Interferon-induced protein 44-like; SAMHD1, Sterile alpha motif and histidine-aspartic acid domain containing protein 1; AUC, area under ROC curve; CI, confidence interval; VR, virological response; SR, serological response; Bold values are statistically significant P < 0.05. | | | | | | | | | | |
