# Supplementary material for: Dynamic changes in IFI44L and SAMHD1 mRNA levels in PBMCs are associated with treatment response in HBeAg-negative chronic hepatitis B during peg-IFN-α therapy
Source: Front Microbiol. 2026 Sep 25;17:1976096. doi: 10.3389/fmicb.2026.1976096 (PMC13649976; doi:10.3389/fmicb.2026.1976096)
Supplement: Supplementary file 8 [file Table_8.DOCX]

**(1) Peripheral blood collection and PBMC extraction**

a. In this study, peripheral blood samples were collected using vacuum blood collection tubes containing EDTA as an anticoagulant. Each sample volume was 5 mL, which was equally divided into two tubes. After collection, the samples should be immediately stored at 4°C and processed within two hours of collection to ensure the stability of the blood samples and the accuracy of the analysis.

b. The plasma separation process is as follows:

a) The biosafety cabinet was disinfected with ultraviolet (UV) light to ensure sterile conditions during the operation, with disinfection lasting for 30 minutes.

b) While disinfection was underway, the peripheral blood samples were placed in a centrifuge, set to a temperature of 4°C, a speed of 1200 rpm, and centrifuged for 10 minutes to separate the plasma.

c) After disinfection was completed, the blood collection tubes were opened, and the supernatant was carefully extracted under sterile conditions inside the biosafety cabinet.

d) The extracted supernatant was subjected to a second centrifugation step at 4°C, 3000 rpm, for 15 minutes to further purify the plasma components.

e) After the second centrifugation, the supernatant was collected and transferred to sterilized EP tubes, with 500 μL aliquots in each tube, which were then stored at -80°C for future analysis.

c. Peripheral blood mononuclear cell (PBMC) extraction process:

a) The peripheral blood samples were diluted with 1×PBS solution at a 1:1 ratio, mixed thoroughly, and distributed into two tubes.

b) During distribution, 4 mL of human peripheral blood lymphocyte separation solution (Ficoll) was gently added to the bottom of the sample tube using a capillary pipette to ensure that the liquid ascended slowly, forming a clear layered structure. The sample was then centrifuged at 400 g for 40 minutes at 20-24°C.

c) After centrifugation, the layered liquid was removed, and the middle layer (the white granular layer) was carefully transferred to a new EP tube.

d) The above steps were repeated to ensure effective recovery of the PBMC layer.

e) The extracted PBMCs were mixed with the pre-prepared cryopreservation solution (RPMI1640 medium + 10% FBS + 50% 2× serum-free cell cryopreservation solution). After cell counting, it was ensured that each tube contained more than 3×10^6 cells, and the cells were aliquoted into 1.5 mL cryovials.

f) The cryovials were placed in a gradient freezing box and stored at -80°C for freezing. After 24 hours, they were transferred to a liquid nitrogen tank for long-term storage to maintain cell viability.

**(2) Extraction of total RNA from cells**

The RNA extraction procedure using the Trizol method was carried out in a biosafety cabinet, using a 6-well plate as an example. The process is as follows:
a. Prior to the experiment, pre-cool the centrifuge to 4°C to ensure that samples are processed under low temperature conditions, preventing RNA degradation.
b. After discarding the culture medium, wash the cells twice with PBS buffer to remove residual medium components and aspirate the PBS.
c. Add 1 mL of Trizol reagent to each well, gently mix to ensure it completely covers the cell layer, and let it stand for 3 to 5 minutes to complete cell lysis.
d. Transfer the lysed cell suspension to a 1.5 mL RNase-free EP tube, use a pipette to mix until the precipitate is completely dissolved, and let it stand at room temperature for 5 minutes.
e. After adding chloroform, gently invert the tube several times to mix, ensuring complete emulsification of the chloroform. Then, allow the tube to stand at room temperature for 5 minutes to allow phase separation.
f. Centrifuge the mixture at 4°C, with a speed of 12,000 g for 15 minutes to separate the different phases.
g. Carefully collect the clear upper aqueous phase and transfer it to a new RNase-free EP tube, add isopropanol, and invert the tube several times to mix. Let it stand at room temperature for 10 minutes to precipitate the RNA.
h. Centrifuge again at 4°C with a speed of 12,000 g for 10 minutes to further separate the precipitate.
i. Discard the supernatant, add 75% ethanol, and gently invert the tube to wash the RNA pellet, taking care not to disturb the pellet, to ensure its purity.
j. Centrifuge at 4°C with a speed of 7,500 g for 5 minutes to further wash and pellet the RNA.
k. Discard the supernatant, allow the pellet to air dry in a ventilated area, and after it is completely dry, dissolve the RNA in 10 to 20 µL of DEPC water. Store the solution at 4°C for future use.

**(3) Reverse Transcription of RNA to cDNA**

According to the instructions of the PrimeScript™ RT Reagent Kit with gDNA Eraser, perform the following steps:
a. To remove genomic DNA, prepare the reaction mixture on ice according to the specified components. It is recommended to prepare 2 times the amount needed for the required number of reactions to ensure sufficient reagents for the reaction.

| **Reagent** | **Dosage** |
| --- | --- |
| 5×gDNA Eraser Buffer | 2.0ul |
| Total RNA | 1ug |
| Rnase Tree ddH_2_0 | add to 10ul |
| gDNA Eraser | 1.0ul |

b. The prepared reaction mixture should be allowed to sit at room temperature for 5 to 10 minutes until it reaches the desired temperature for the experiment.
c. The reverse transcription reaction should be performed on ice. To ensure the accuracy and stability of the reaction, the reagents should be pre-prepared in an amount that is 2 times the required number of reactions. Then, transfer 10 µL of the mixture into each reaction tube, gently mix to ensure even distribution of the reagents, and then begin the reverse transcription process.

| **Reagent** | **Dosage** |
| --- | --- |
| Reaction solution of step a | 10.0ul |
| PrimeScript RT Enzyme Mix I | 1.0ul |
| 5×PrimeScript Buffer II | 4.0ul |
| Rnase Tree ddH_2_0 | 4.0ul |
| RT Prime Mix | 1.0ul |

d. The reverse transcription reaction should be performed in a PCR machine with the following conditions: 37°C for 15 minutes → 85°C for 5 seconds → 4°C for 1 minute.
e. Store the cDNA at -20°C.

(3) Amplifying the target gene
Follow the instructions of the SYBR Green qPCR Master Mix kit. Perform the entire process on ice, and prepare the reaction mixture according to the following components:

| **Reagent** | **Dosage** |
| --- | --- |
| SYBR Green | 5.0ul |
| FP（10uM） | 0.2ul |
| RP（10uM） | 0.2ul |
| cDNA | 1.0ul |
| ddH_2_0 | 3.6ul |

Use the Bio-Rad fluorescence quantitative PCR instrument CFX96 to perform real-time fluorescence quantitative PCR on cDNA with the following conditions:

- Pre-denaturation: 95°C for 30 seconds
- PCR: 95°C for 5 seconds; 60°C for 30 seconds × 40 cycles
- Melting: 95°C for 5 seconds; 60°C for 1 minute; 95°C
- Cooling: 50°C for 30 seconds
- Read fluorescence signal.
